# Cyclo- and Polyphosphazenes for Biomedical Applications

**DOI:** 10.3390/molecules27238117

**Published:** 2022-11-22

**Authors:** Girolamo Casella, Silvia Carlotto, Francesco Lanero, Mirto Mozzon, Paolo Sgarbossa, Roberta Bertani

**Affiliations:** 1 Department of Earth and Marine Sciences (DiSTeM), University of Palermo, Via Archirafi 22, 90123 Palermo, Italy; 2 Department of Chemical Sciences (DiSC), University of Padova, Via F. Marzolo 1, 35131 Padova, Italy; 3 Institute of Condensed Matter Chemistry and Technologies for Energy (ICMATE), National Research Council (CNR), c/o Department of Chemical Sciences (DiSC), University of Padova, Via F. Marzolo 1, 35131 Padova, Italy; 4 Department of Industrial Engineering, University of Padova, Via F. Marzolo 1, 35131 Padova, Italy

**Keywords:** cyclophosphazenes, polyphosphazenes, drug delivery, tissue engineering

## Abstract

Cyclic and polyphosphazenes are extremely interesting and versatile substrates characterized by the presence of -P=N- repeating units. The chlorine atoms on the P atoms in the starting materials can be easily substituted with a variety of organic substituents, thus giving rise to a huge number of new materials for industrial applications. Their properties can be designed considering the number of repetitive units and the nature of the substituent groups, opening up to a number of peculiar properties, including the ability to give rise to supramolecular arrangements. We focused our attention on the extensive scientific literature concerning their biomedical applications: as antimicrobial agents in drug delivery, as immunoadjuvants in tissue engineering, in innovative anticancer therapies, and treatments for cardiovascular diseases. The promising perspectives for their biomedical use rise from the opportunity to combine the benefits of the inorganic backbone and the wide variety of organic side groups that can lead to the formation of nanoparticles, polymersomes, or scaffolds for cell proliferation. In this review, some aspects of the preparation of phosphazene-based systems and their characterization, together with some of the most relevant chemical strategies to obtain biomaterials, have been described.

## 1. Introduction

Phosphazenes are interesting and versatile chemical substrates characterized by the presence of -P=N- repeating units giving rise to low-molecular-weight cyclic structures with three or four units up to polymers containing thousands of -P=N- moieties, where the P atom in the starting material (i.e., hexachlorocyclotriphosphazene, HCCP) bears two chlorine atoms [1].

**Scheme 1:**
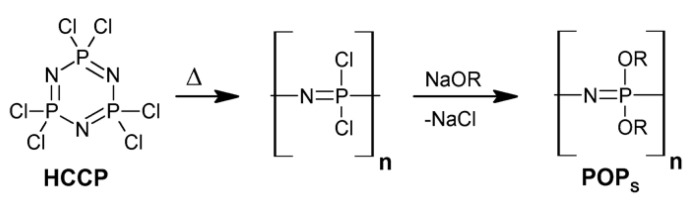
From hexachlorocyclotriphosphazene (HCCP) to polyphosphazenes (POPs).

**Scheme 2:**
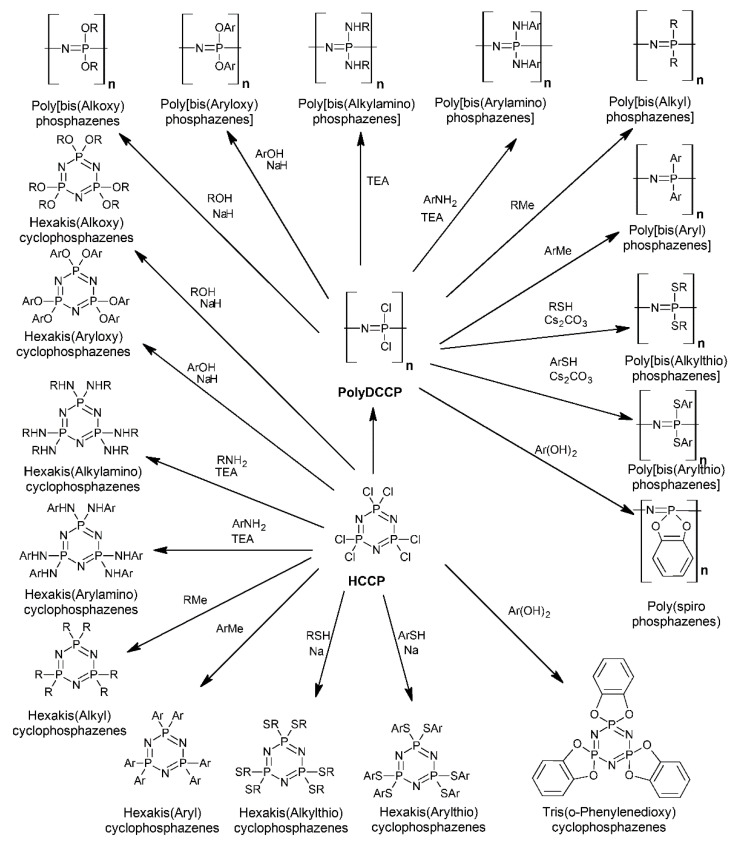
From HCCP and polydichlorocyclophosphazene (PolyDCCP) to a wide variety of derivatives by nucleophilic substitution.

The chemistry and the properties of phosphazenes, in view of industrial applications, have been reviewed in a series of books [2,3] and articles [4] stemming from papers published in 1964–1965, when Allcock and coworkers [5,6,7] first reported the synthesis of linear poly(organophosphazenes) (POPs) through the thermal-induced ring-opening of the HCCP and the subsequent substitution of the chlorine atoms with suitable organic groups to achieve a wide variety of new derivatives (Scheme 1 and Scheme 2).

The scientific and applicative interest for phosphazenes arises from the relatively easy substitution of the chlorine atoms with an enormous variety of substituents, thus giving rise to an extremely wide number of new materials whose properties can be designed, in principle, based on the nature of the substituents in addition to the specific characteristics of the -P=N- backbone. Phosphorus is one of the most important elements preventing the combustion of organic materials, with a synergistic effect of nitrogen. Thus, not only is the -P=N- backbone nonflammable but it also quenches the combustion of other compounds in contact with it, likely due to both the interruption of the free radical processes and the formation of an intumescent barrier to the oxygen entrance [8,9]. Furthermore, the nature of the P-N bond guarantees an extremely low torsion barrier of the backbone, thus showing glass transition temperatures of some polyphosphazenes in the −100 °C region [10,11].

It is noteworthy that a lot of patents have been deposited over time based on phosphazenes, exhibiting specific properties of industrial interest. The most intriguing properties, which can be modulated on the bases of the molecular weight, structure, nature, and combination of substituents, range from the thermal resistance of the polymers, the tuneable low-glass-transition temperature, the hydrophilic/hydrophobic behavior and the water/solvent solubility to the compatibility with inorganic materials, owing to the possibility to introduce organosilicon moieties as substituents [11], and the formation of aerogels with various densities by the crosslinking of cyclotriphosphazenes and polysiloxanes [12].

Cyclophosphazenes have been proposed as hydraulic fluids, lubricant stabilizers and additives, in particular with trifluoromethylphenoxy substituents [3], as substrates for supramolecular assemblies [13,14,15], and as supports for metal catalysts, either through metal coordination by the nitrogen atoms of the backbone or through the presence of ligands as substituents [11,16,17].

Upon the polymerization and substitution of the reactive chlorine atoms, they give rise to a wide variety of new polyphosphazenes, containing from 100 to 15,000 or more repeating units (with molecular weights ranging from 2 to 10 × 10^6^ Da) with an unusually broad range of useful properties. Polyphosphazenes with elastomeric [18,19], optical [20], proton-conducting [21], electrochemical [22], and fire-resistant [23] properties have been investigated and applied in the development of membranes [24], fuel cells [25], and hybrid materials [12]. New elastomeric inorganic silicon-based compounds, and specifically fire-resistant elastomers and plastics, have been studied for military purposes [26,27].

Due to the huge number of phosphazene applications, we decided to delimit the topics of this review, focusing our attention on the intriguing results of the investigations on phosphazene systems in the biomedical field. Thus, we gathered the scientific literature published after the books edited by Andrianov in 2009 [15] and by Teasdale [28] with the aim to collect the most fascinating aspects of the chemistry of these materials based on their synthetic versatility. The patents in the field have not been considered.

## 2. Synthesis and Characterizations

### 2.1. The Syntheses and the Architectures

Cyclic phosphazenes [NPCl_2_]_n_, with n < 20, are classically prepared by reaction of PCl_5_ with NH_4_Cl in a high-boiling chlorinated solvent (i.e., tetrachloroethane, 132–145 °C, 6–20 h) followed by the rapid distillation of the solvent, separation from the rubber-forming higher polymers, a final fractionation of the trimeric and tetrameric compounds, which are the major products, and purification through recrystallization and sublimation [29,30]. The effects of different reaction conditions have been investigated, even if the reaction mechanism remains difficult to interpret. Reasonably, the reaction proceeds through the formation of NH_4_PCl_6_, which decomposes to NH=PCl_3_, which then polymerizes with the elimination of HCl or reacts with PCl_5_ [31].

NH_4_Cl + PCl_5_ → NHPCl_6_ → NH=PCl_3_ → (NPCl_2_)_n_

**Figure 1:**
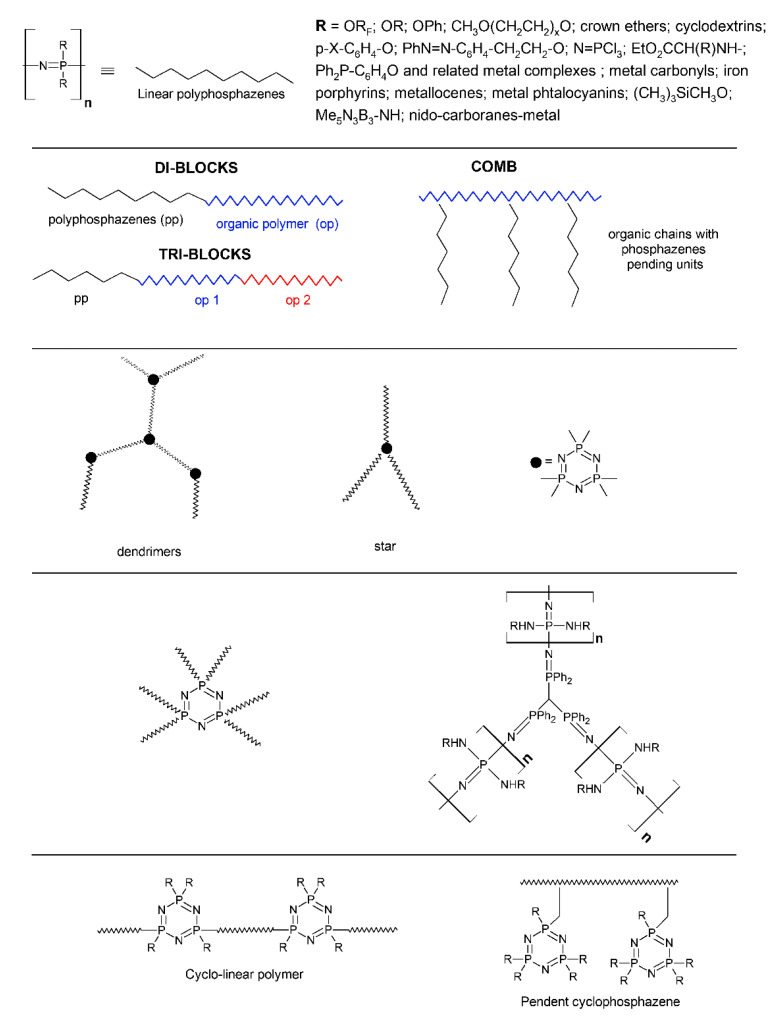
The different frameworks of the P=N backbone.

As summarized in Figure 1, different frameworks can be achieved based on the -P=N- backbone: cyclic structures, linear polymers, copolymers [11,15,32,33,34,35] (regular or random diblock or triblock copolymers), combs, stars with or without a cyclophosphazene core, dendrimers, cyclolinear and cyclomatrix polymers, and polymers with pendent cyclotriphosphazene rings.

**Figure 2:**
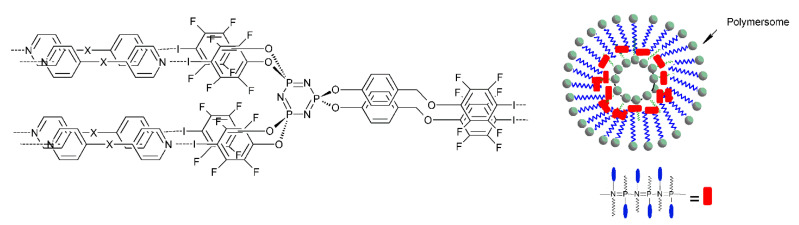
Some supramolecular arrangements of phosphazenes: tapes and polymersomes.

The availability of a wide range of organic substituents on the -P=N-skeleton also allows the achievement of different 3D architectures in supramolecular arrangements, from electrospun scaffolds [36] to micelles and polymersomes [37], when amphiphilic moieties are bonded to the P atoms or through noncovalent interactions (Figure 2) [38,39,40].

Different strategies have been proposed and optimized to produce polymers with substituents not easily available and to give different structures [41].

**Scheme 3:**
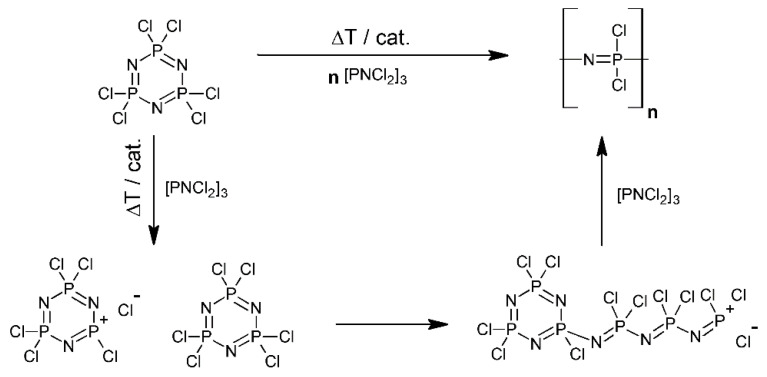
Proposed mechanism for the cationic chain–growth polymerization process.

(i) Thermal-ring-opening method: from HCCP at 250 °C under a vacuum through a cationic chain-growth polymerization process, due to the formation of the cationic species [P_3_N_3_Cl_5_]^+^ by chlorine loss [41], which initiates the opening of a second ring, thus propagating the polymerization (Scheme 3).

**Scheme 4:**
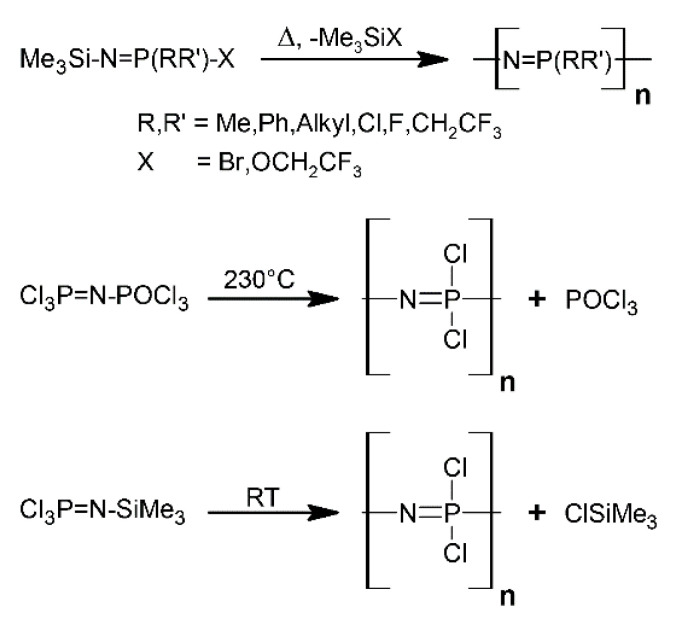
Some living polymerization processes.

**Scheme 5:**
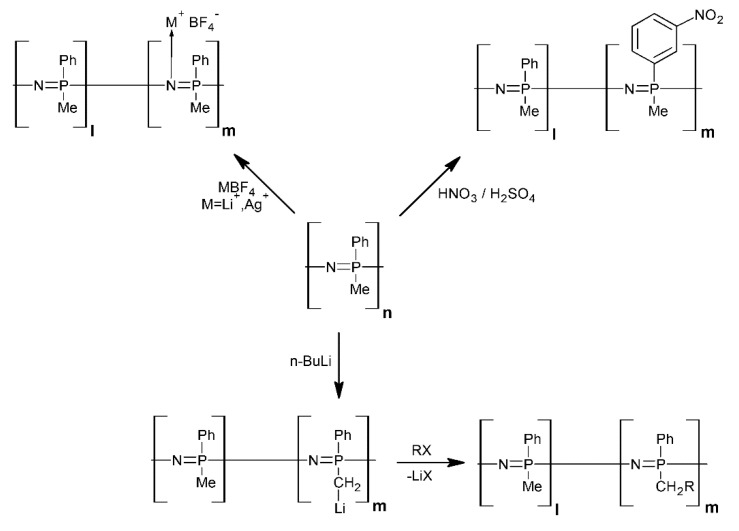
Some reactions carried out on –[P(Me)(Ph)=N]_n_–polyphosphazenes.

A drawback of the nucleophilic substitution of the chlorine atoms in the P=N backbone is that all reactions cannot reach 100% substitution of chlorine atoms. As a consequence, the unreacted P-Cl quickly reacts with moisture, giving P-OH moieties and leading to uncontrolled crosslinking and degradation, thus compromising the properties of the final polymers. To achieve control over the molecular weight and polydispersity, various catalysts have been used [42].

(ii) Living cationic polymerization method by the reaction of (Cl3P=NSiMe3) with PCl5 [43]. An intriguing study concerning the mechanism of the ambient temperature PCl5-initiated living cationic chain growth polycondensation of Cl3P=NSiMe3 provided evidence that, under the usual polymerization conditions, the propagation occurs at both chain ends and identified factors to potentially control the molecular weight and broadening of the molecular weight distribution [44]. It has been observed that good control over the molecular weight and polydispersity can be achieved for short polymer chains (up to 50 units), while in the case of longer polymer chains, a lower control can be obtained. Detailed kinetic studies have been carried out to investigate the mechanism of the reactions and optimize the polymerization conditions (Scheme 4) [42,45,46]. A wide variety of reactions, from enhancing the basicity of the backbone N atoms to the electrophilic substitution on the phenyl ring or the exploitation of the relative acidity of the P-CH3 groups for the formation of carbanions, which can react with a wide variety of electrophiles, have been investigated (Scheme 5).

For R = Ph and R’ = Me, many subsequent reactions can be carried out (Scheme 5) [47].

**Figure 3:**
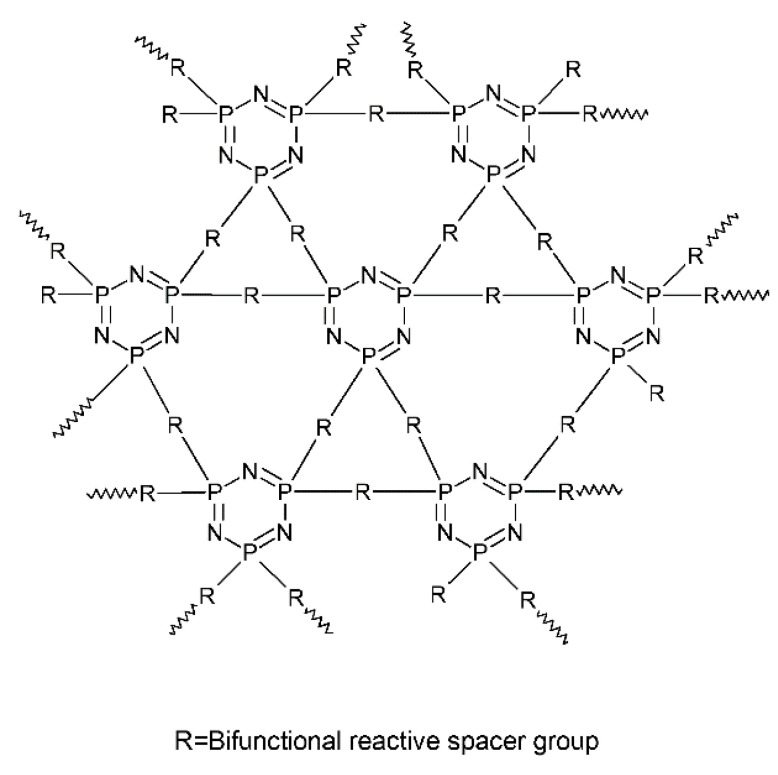
Schematization of a cyclomatrix polyphosphazene.

Of note is that phosphazene moieties bearing π-donating dialkylamino substituents bonded at phosphorus have been reported to be Bronsted superbases, with an excellent delocalization of the engendered positive charge of the cations, thus being able to deprotonate weakly acidic compounds [48,49].

(iii) Synthesis of cyclomatrix polyphosphazenes, through the chlorine substitution with bifunctional reactive spacer groups, such as diamines or diols, which can give rise to different self-assembled geometries (microspheres, hollow spheres, nanotubes, nanofibers, and sheets) (Figure 3) [3,50,51].

### 2.2. The Characterization

**Figure 4:**
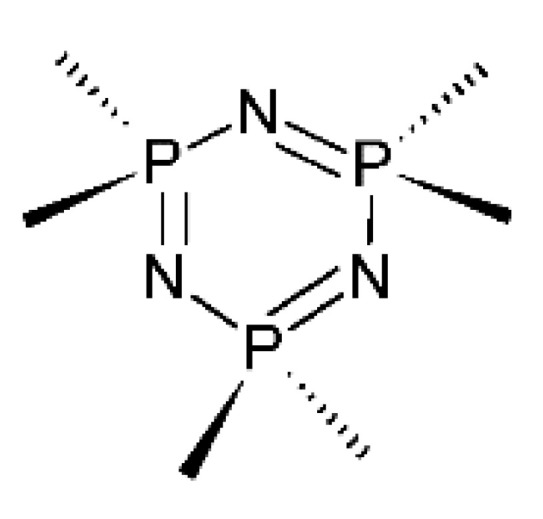
Arrangement of the substituents on the P atoms of the cyclotriphosphazene ring.

Cyclotriphosphazenes feature a nearly planar ring structure that provides a rigid D_3h_ symmetrical support for the six P-bonded substituents arranged on opposite sides with respect to the plane of the N=P cycle and outside, as shown in Figure 4 [17].

Changes in the molecular parameters of cyclophosphazenes have been studied as a function of substituents at fixed positions, observing specific trends. As an example, the progressive substitution of chlorine atoms in the HCCP structure with HNBu^t^ moieties increases the P-N bond lengths involving the P atom bearing the substituents and the remaining P-Cl bonds [52].

**Figure 5:**
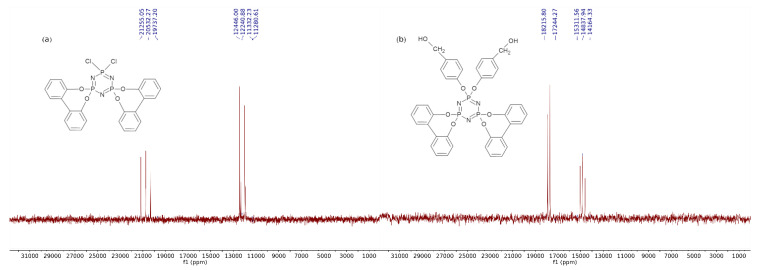
^31^P NMR spectra in CD_3_COCD_3_ of (**a**) = 2,2–dichloro–4,4,6,6,-bis[spiro(2′,2″–dioxi–1′,1″–biphenyl)]cyclotriphospazene and (**b**) = 2,2–bis(4–formylphenosy)–4,4,6,6,–bis[spiro(2′,2″–dioxi–1′,1″–biphenyl)]cyclotriphospazene.

Spectroscopically, the presence of intense absorptions at about 1200 cm^−1^ (P-N-P stretching mode) and at 700–950 cm^−1^ in the FTIR spectra of the compounds indicates the presence of the phosphazene ring. The ^31^P NMR technique allows investigating the number, the nature, and the position of the substituents, starting from the singlet at 21.23 ppm (in CD_3_COCD_3_) for HCCP [53,54] until the characterization of dendrimeric structures (Figure 5) [3].

Depending on the number and position of substituents, up to three chiral centers can be achieved, giving rise to racemic and diastereoisomers species. Many investigations have been carried out, mainly on >P(spiro) systems bearing a bidentate moiety on two of the P atoms and different ones on the third (Figure 5) [55].

As for polyphosphazenes, their stereogenic properties attracted a great deal of attention in polymer science, as well as biological, pharmaceutical, and medicinal science, because of their high potential for application in advanced materials. In the case of linear [N=P(RR’)]_n_ polymers, the phosphazene moiety has a chiral center and the polymer is stereogenic. In the case of cyclolinear and cyclomatrix polymers, the stereogenic properties depend on whether there is more than one type of substituent in the “monomer” unit. These polymers can be optically active, giving rise to meso, racemic, or diastereoisomeric mixtures. Again, ^31^P NMR studies can help the understanding of the structure and the properties of these systems [56,57].

### 2.3. Computational Approaches to Phosphazenes

In this section, we will briefly describe the computational approaches to the description of the P-N bond in phosphazenes, as well as the Molecular Dynamics (MD) methods to deal with macrosystems, such as the polyphosphazenes employed in biochemical systems.

#### 2.3.1. Quantum Chemical View of the P-N Bond in Phosphazenes

Substituted cyclotriphosphazenes (Scheme 1 and Scheme 2), often in the relevant HCCP form, and polyorganophosphazenes (POPs) represent the building blocks of a huge substitution chemistry.

**Figure 6:**
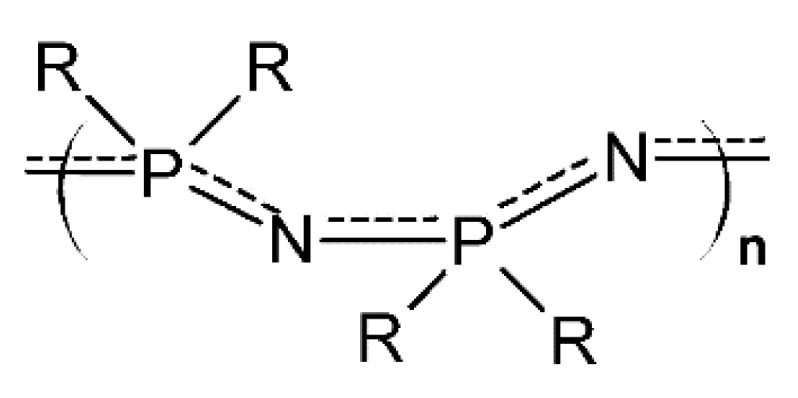
Configuration of the most stable cis,trans-POP isomer.

The nature of the P-N bond in these inorganic systems is a matter of long debate. The P-N bond length in phosphazenes is about 1.60 Å, while related saturated phosphazenes show a P-N bond length of about 1.80 Å [53]. Moreover, the cyclotriphosphazene ring is planar without bond length alternation. From the standpoint of the valence bond (VB) theory, the bond in the ring in cyclotriphosphazene should have a multiple bond character and the presence of six π valence electrons should also confer aromaticity, thus further stabilization, to the ring. Incidentally, this picture has been rationalized by Dewar in the so-called “island model” [58], where the Pdπ-Npπ overlap causes electron density “islands” along the P-N-P units with nodes on the P atoms. Concerning the POPs, also in this class of compounds, the P-N bonds present characteristics almost alike to cyclotriphosphazenes. It is worthy of note that the lack of bond length alternation is not a constant feature of these systems. Indeed, bond alternation has been sometimes observed both for substituted cyclotriphosphazenes and polymers. On these grounds, the quest concerning the nature of the P-N bond in phosphazenes has been focused on specific topics, i.e.,: (i) if dπ of the P are involved in the π electrons delocalization and (ii) if there is ring aromaticity when 4n + 2 π electrons are involved in the delocalization. In this context, it has been shown by charge density, Natural Bond Orbital (NBO), and Energy Decomposition Analysis–Natural Orbitals for Chemical Valence (EDA-NOCV) approaches that, both for cyclic- and polyphosphazenes, the PN bond is highly polar, with a remarkable ionic character [59,60,61,62] along with the presence of a negative hyperconjugation involving the N lone pair with the σ*_PX_ (X = ligand at the P atom) and, to a lesser extent, the σ*_PN_ orbitals [59,60,61,62,63]. These outcomes discarded the hypervalent character of the P due to the participation of P dπ orbitals in the delocalization of π electrons. Moreover, a charge density investigation [63] showed that the cyclophosphazenes show electron density “islands” in chloro tri- and tetracyclophosphazenes, allowing the separation in modular units of Cl_2_PN. These outcomes resemble Dewar’s “island” models where, however, no P dπ orbitals are involved. The P-N bond picture obtained, i.e., a highly polarized P-N bond and Np→σ*_PX_, σ*_PN_ negative hyperconjugation, also satisfactorily explain the P-N bond length alternation, mainly in POP systems. Indeed, it has been shown that the extent of the NBO overlap of the orbitals involved in the hyperconjugation is responsible for the P-N-P angles which, in turn, affect the P-N bond polarity. These outcomes explain the alternating P-N bond in the most stable cis,trans-POP configuration (Figure 6) due to the different NBO overlaps between the cis and trans P-N bond patterns [59,64].

**Figure 7:**
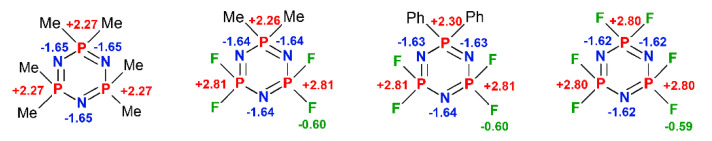
Examples of NBO charges on P and N atoms depending on the nature of substituents on the P atoms in cyclophosphazenes [56]. Of note is the independence of the charge on N from the substituent on P.

The role of the substituents on the properties of the P-N bond has also been investigated by several computational methods, e.g., electron density, NBO, and EDA-NOCV analysis [59,60,62,65]. The donor/acceptor as well as the electron-withdrawing behavior of the ligands bonded to the P modulates the electron density at the P center without affecting the electron density on N. Thus, a withdrawing group causes an electron density depletion at the P and then an increasing P-N polarization, leading to a shortening of the P-N bond and vice versa (Figure 7).

Finally, the aromaticity of the cyclotriphosphazene rings was also investigated. Chaplin et al. [59] cautiously proposed the presence of aromaticity in cyclotriphosphazenes according to the Nucleus-Independent Chemical Shift (NICS) analysis. Indeed, the seminal NICS(0) and NICS(1) descriptors may also be affected by the contribution of currents, leading to erroneous conclusions [66]. Experimental charge density studies on the HCCP definitely assessed the lack of pseudoaromatic delocalization. Moreover, the very-high-similarity behavior of the P-N bond in HCCP and octachlorocyclotetraphosphazene enforced the guess that the ring planarity in cyclotriphosphazenes represents the more stable conformation [63].

#### 2.3.2. Molecular Dynamic Simulations of Phosphazenes

One of the peculiar characteristics of POPs which entitle them as ideal candidates for a wide variety of industrial applications is due to the flexibility of the backbone due to the P-N bonds. Indeed, this bond favors the membrane formation in biological environments, and many substituent groups, bonded to the phosphorus, can easily modulate the hydrolytic instability and consequently the biodegradability [15]. With respect to this habit, the molecule dynamic (MD) simulations allow for gaining important insight into the molecular properties of these polymers and establishing the structure–property relationships. Despite their usefulness, only a few numbers of MD studies are reported in the literature and are often limited to small oligomers that do not allow for the reproduction of the behavior of long-chain polymers [67,68,69]. About 20 years ago, some studies demonstrated that a modified CHARMm [68,70,71], AMBER [72], or DREIDING [73] force field can reproduce the geometrical parameters (bond lengths, valence, and torsional angles) in polyphosphazenes with almost 20 repeat unit chains. The COMPASS force field was used since 1998 by Fried et al. [74,75,76,77,78] to consider also the condensed-phase properties such as the glass transition temperature, diffusion coefficients, and density. In more recent years, Kroger and Fried focused their attention on polyphosphazenes for biomedical applications [79]. Their MD simulations allow them to determine the bulk properties and investigate the atomic interactions. They correlate the hydrogen bonds with the electrostatic interactions and the solubility of the polymers. All these aspects are crucial when these polymers are used to control drug release [80], in drug delivery applications [81,82], or as a microencapsulation material, as well as an immunoadjuvant [15]. In very recent work, Wang et al. rationalized the effect of the side groups on the glass transition polymers starting from a system with 150 repeat units [83], but a great advance in the MD simulations has been made by Chen et al. [84]. In this study, the DREIDING force field with the Lennard–Jones potential was used, and they obtained an in situ dynamic polymerization procedure to make, test, and tune the thermos-mechanical properties of polyphosphazenes via MD simulations, which was obtained. The bonds between monomers were formed during the simulations and this allowed, for example, to consider the different end-to-end polymer interactions that influence the macroscopic properties. Moreover, differently from the previous models, a dynamic procedure was more versatile, and the properties of the POPs could be tested also as a function of the degree of polymerization and not on fixed repeat units.

## 3. Biomedical Applications

**Scheme 6:**
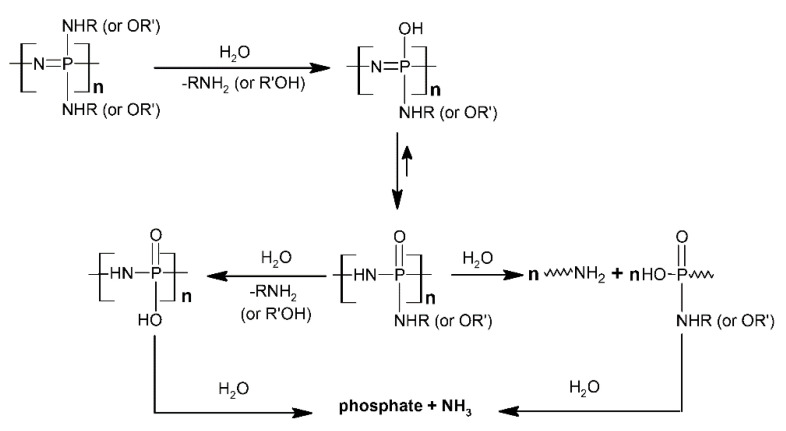
Hydrolytic degradation mechanism of POPs.

During the last 20 years, a wide variety of new phosphazene systems, either trimeric or polymeric, have been developed as biomaterials in view of different applications. The materials to apply in biomedical applications should be biocompatible, in other words nontoxic themselves, as well as their degradation products. Moreover, both the materials and their degradation products also should not induce an inflammatory, carcinogenic, pyrogenic, or allergic response. The degradation products of many polyphosphazenes form a buffering system (ammonium phosphate) and maintain a neutral pH through the degradation [85,86]. The most important advantage of polyphosphazenes over other polymers is the possibility to introduce side groups with specific chemical–physical and biological behavior to design biomaterials for tailored applications. Fluorinated chains improve the hydrophobicity, giving rise to materials suitable for surface modifications, and have been approved as dental liner materials because of their antimicrobial properties and biological inertness [15,87]. The introduction of amino acid esters improves the degradation of the polymers: the backbone degradation gives rise to nontoxic products (phosphate and ammonia) and can be affected significantly by the presence of residual chlorine atoms and hydroxyl groups along the chain, together with the introduction of hydrolytically labile side groups: a number of amino acid esters have been introduced, also in combination, observing that their steric hindrance can modulate the hydrolysis rate. The materials can be used for drug delivery, tissue engineering, or shape–memory polymers for cardiovascular or bile duct stents, as examples, where the material can be either biostable or biodegradable into nontoxic end-products according to a modulable designed degradation rate (i.e., such as the rate of tissue growth or according to a desired therapeutic release rate) [42,88]. It has been observed that the presence of only small amino acids as substituents such as glycine and alanine induced a quicker degradation than phosphazenes bearing larger or phenoxy substituents. The modulation of the degradation rate could also be obtained with a combination of hydrophilic and hydrophobic side groups (i.e., carbohydrates or steroidal substituents) [89,90,91,92]. The degradation mechanism involves the attack of water molecules on organic side groups on the POPs, with the formation of P-OH units by the migration of protons from oxygen to nitrogen, thus sensitizing the polymer backbone to hydrolysis, yielding nontoxic degradation products which comprise mainly NH_3_, phosphate, and the corresponding side groups, as depicted in Scheme 6 [93,94].

**Scheme 7:**
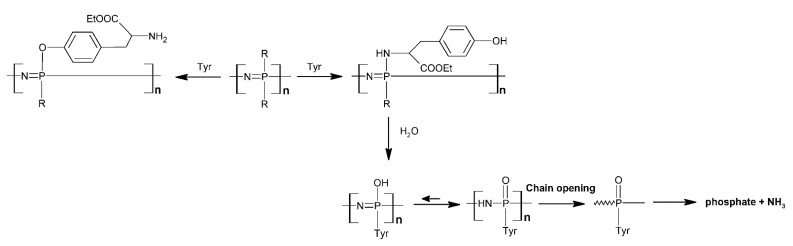
Degradation process of the tyrosine substituted POP.

It was observed that the degradation rates in polymers with side groups linked through the N-atom or the O-atom are different [95]. In the case of tyrosine, which can be attached to the polymer backbone either by amino or by phenolic moiety, only the polyphosphazene-bearing N-tyrosine side groups are biodegradable, while the phenolic group makes the polymers nondegradable but pH-sensitive (Scheme 7) [96,97,98,99]. The degradation rate of some water-soluble polyphosphazenes (bearing amino acid ester units, or pyrrolidinyl, or carboxylatophenoxy moieties) has been studied as a function of the pH, observing a considerably faster degradation at lower pH values. The hydrolytic stability can be tailored by the careful choice of the amino acid spacer and increased by the steric shielding of the polymeric backbone [100,101,102].

The introduction of functionalities into phosphazene pendant groups allows for the attachment of specific molecules into the system that can increase the affinity for the desired species. An example could be aminoethoxyethanol: the oxygen atom can be bonded to P and the amino unit can be used to bind, for instance, galactose or polyethylene glycol moieties, giving rise to materials able to interact with DNA [103,104].

As for tissue engineering applications, other properties must be explored:

(i) Glass transition temperature compared with the physiological temperature: as for bone tissue engineering, a glass transition temperature higher than the physiological one to maintain structural integrity in an in vivo environment is required [105];
(ii) Mechanical properties: substituents must be chosen in order to match the mechanical properties of the POPs (compressive and tensile strengths) and those of the native tissues;
(iii) Porosity and porous interconnectivity of biomaterials plays a key role either in drug delivery applications, due to their controlled degradability, or in tissue engineering, aging as materials scaffolds for cells proliferation;
(iv) Stimuli-responsive site behavior: temperature, ultrasound, light, pH, ionic strength, oxidative conditions, and enzyme presence are important stimuli for biomedical applications. Several stimuli-responsive materials have been prepared for tissue engineering and drug delivery due to the possibility of tuning the properties from combinations of different side groups [105,106,107,108]. The reaction of hexakis [4-(acrylamido)phenoxy]cyclotriohosphazene] with N-isopropylacrilamide and N-vinyl imidazole in the presence of ammoniumpersulfate gave crosslinked hydrogels which exhibited in vitro pH-responsive drug-release behavior [107].

In a quite recent review [109], the opportunity to combine the benefits of an inorganic backbone and a wide variety of organic (or organometallic) side groups in POPs have been considered for future bioapplications, such as the use of cyclomatrix polyphosphazenes to encapsulate particles suitable for imaging applications [110] or to apply POPs in a prototype of an artificial heart [111].

### 3.1. Phosphazenes in Drug Delivery

#### 3.1.1. Biological Activity of Cyclophosphazenes

The substitution of the chlorine atoms in trimers or tetramers with N-monodentate, N-N, or N-O bidentate moieties gave the formation of a series of phosphazene systems for which the antimicrobial activity against Gram-positive and Gram-negative pathogenic bacteria and fungi was tested.

**Scheme 8:**
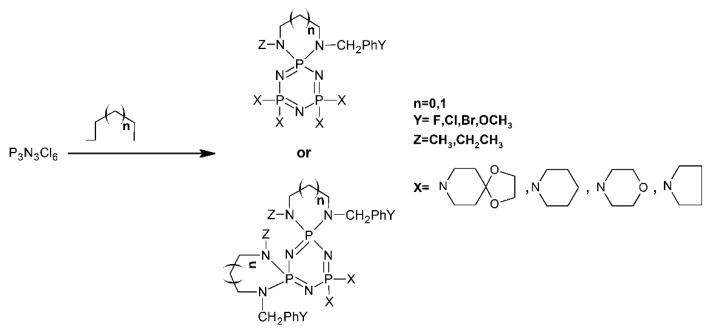
Cyclotriphosphazenes bearing N-N bidentate moieties with antibacterial activity.

**Figure 8:**
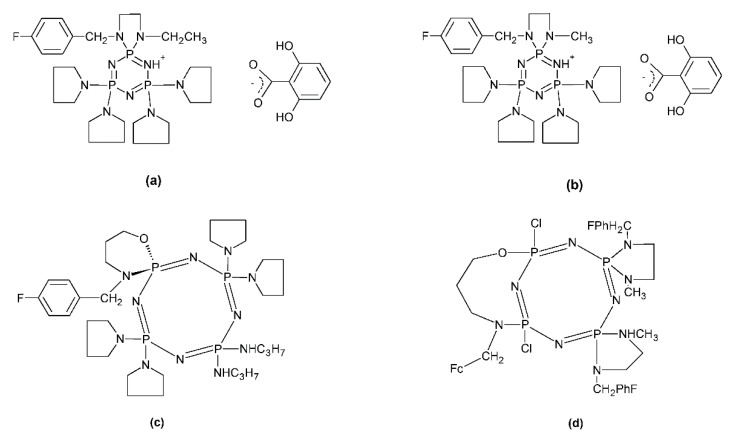
(**a**,**b**): Protic phosphazene salts; (**c**,**d**) Neutral cyclotetraphosphazenes. (Fc=Ferrocenyl).

The tetrapyrrolidino derivatives reported in Scheme 8 were found to inhibit the bacteria *E. coli* ATCC 25922, *P. aeuroginosa*, *B. cereus*, and *P. vulgaris* in a comparable extent as control antibiotics and demonstrated to be more active than Ketokonazole against *C. albicans* and *C. tropicalis*, exhibiting higher activity than the analogue PCl_2_-derivatives [112,113]. It has been demonstrated that the interaction of cyclophosphazenes with DNA caused a decrease in the mobility and intensities of form I and form II DNA due to the binding of the compounds with DNA to A/A and G/G nucleotides [114,115,116]. Similar compounds have been converted to protic salts, as reported in Figure 8, for which an antiproliferative effect on tumor cell lines (A549, Hep 3B and FL) higher than both 5-fluorouracile and cisplatin was demonstrated, the most active being the compounds (**a**) and (**b**) [117].

**Figure 9:**
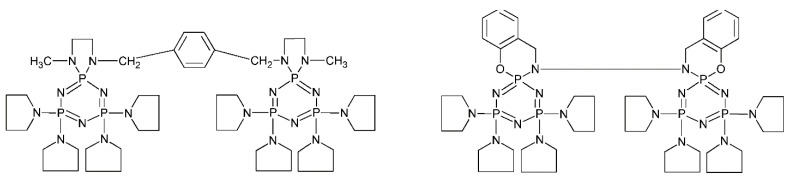
Dimeric cyclophosphazenes with biological activity. The compound on the left also exhibited high cytotoxicity against fibroblast cells; the compound on the right was found to be active against yeast strain *C. tropicalis*.

Tetramers similar to compound (**c**) showed greater inhibitory activity against *K. Pneumonia*, *C. tropicalis*, and *C. albicans*, and in the latter case, more efficient than Ketokonazole [118,119]. Additionally, trimers and tetramers bearing the N/O donor-type bidentate ligands containing a mono-ferrocenyl group have been shown to demonstrate antituberculosis and cytotoxic activity [120,121,122]. Some ansa-spiro cyclotriphosphazenes have been synthesized [123] which exhibited antimicrobial activity against bacteria, one yeast strain, and cytotoxic, apoptotic, and necrotic effects against L929 fibroblast and A549 lung cancer cells. The biological activity of mono-ferrocenyl-2-*cis*-4-dichloro-ansa and mono-ferrocenyl-spiro-tetracyclophosphazenes has been investigated as well, observing a proliferative effect on L929 fibroblast and MCF7 breast cells up to 200 mg/mL, but a significant antituberculosis effect against the *M. tuberculosis* H37Rv reference strain (compound **d** of Figure 6) [124]. Dimeric cyclophosphazenes have been achieved by the reaction of HCCP with symmetric N_2_N_2_ or N_2_O_2_ tetradentate donor ligands: antibacterial activity against Gram-positive and Gram-negative bacteria has been observed. In Figure 9, the compound on the left also exhibited high cytotoxicity against fibroblast cells, while the compound on the right was found to be active against yeast strain *C. tropicalis* [125,126].

**Figure 10:**
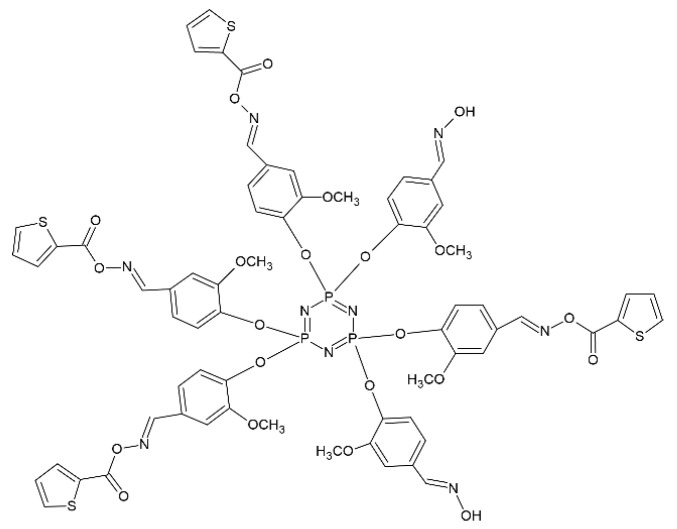
The oxime cyclotriphosphazene derivative exhibiting antimicrobial activity comparable to Ciprofloxacin and Fluconazole.

A series of cyclophosphazenes bearing oxime groups as substituents have been reported [127] to exhibit a significant antimicrobial activity against Gram-positive (*S. aureus* and *E. faecalis*) and Gram-negative (*E. coli* and *K. pneumoniae*) microbes. In particular, the cyclotriphosphazene bearing four thiophene-2-carbonyl derivatives reported in Figure 10 also showed antifungal activity (against *A. niger* and *C. albicans*).

To improve the antimicrobial activity, AgL complexes (L = PPh_3_, PPh_2_Me) have been bonded to N-ring atoms of the cyclophosphazene to achieve the compound [N_3_P_3_(NHCy)_6_{Ag(PPh_2_)}_3_](TfO)_3_, which showed a significantly higher antitumor activity against MCF_7_ and HepC2 cell lines compared to cisplatin and very low MIC (μM) values against *S. aureus*, *M. bovis (BCG)*, and *M. tuberculosis(H37Rv)* [128].

**Figure 11:**
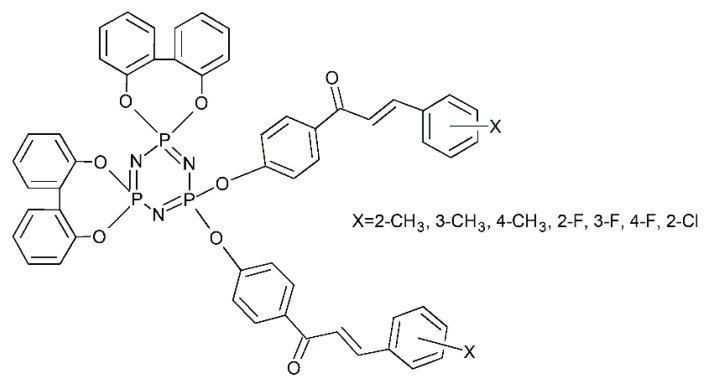
Chalcone-cyclophosphazene derivatives.

Molecular docking studies showed that cyclophosphazene systems bearing 4-oxyphenyl-3-(substituted-phenyl)prop-2-en-1-one [129] and heteroring chalcones [130] arms interact at the tubulin-binding cavity, similarly to colchicine, and with DNA on active sites of Bcl-2, p-53, Caspase-3, and SRC-kinase enzymes, respectively. The chalcone-cyclophosphazene compounds with the structure depicted in Figure 11 have been shown to be active in vitro against human prostate PC-3 and LNCaP cancer cell lines, the most active being the F-substituted derivatives [131].

**Figure 12:**
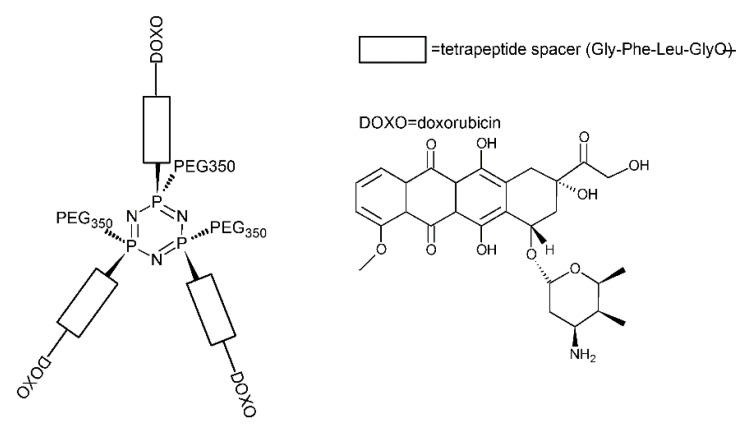
A biodegradable cyclophosphazene prodrug of doxorubicin.

A biodegradable water-soluble cyclotriphosphazene bearing doxorubicin, methoxy-poly(ethylene glycol)_350_ and a tumor-specific tetrapeptide (Gly-Phe-Leu-Gly) have been prepared to study the effect of the enzymatically controlled release on the cytotoxicity against the leukemia L12110 cell line. A lower activity than that of free doxorubicin has been observed but a higher in vitro cytotoxicity, such as cisplatin (Figure 12) [132].

#### 3.1.2. Polyphosphazenes

The tunable degradation rates of polyphosphazenes with the formation of nontoxic products make them largely advantageous for drug delivery applications together with the possibility to design stimuli-responsive frameworks. Polymeric drug delivery systems have been prepared according to two different categories:

(i) To achieve controlled drug release systems where the role of the polymer is to extend the half-time of the drug;
(ii) To achieve targeted drug delivery systems carrying drugs to the sites of action, being usually severely cytotoxic drugs, such as anticancer ones with tumor selectivity [42].

The polymer was designed to perform three different functions in the delivery system owing to the nature of the interactions (H-bonds, π–π, or noncovalent interactions) occurring between the polyphosphazene side-chains and the drug or directly bearing the drugs bonded as substituents on the polymeric chain. Thus, the polyphosphazene can:

(i) Improve interpolymer complexation during the formation of the mixed polyelectrolyte;
(ii) Promote the release of polynucleotides from endolysosomal compartments;
(iii) Reduce polycations caused by toxicity.

Polyphosphazenes bearing polyethylenglycol (PEG)-type arms have been tested for the drug delivery of reference drugs such as platinum derivatives [133]. Doxorubicin and paclitaxel in conventional micelle, hydrogels, or nanoparticles, but also new polymer-drug-conjugated forms have been developed, behaving as prodrugs. Different polyphosphazene systems have been studied to bind Pt(II) systems, such as [NP(PEG_550 or 350_)_x_(GlyGluPt(dach))_2−x_] (dach=1,2-diaminocyclohexane), or to behave as macromolecular Pt(IV), ruthenium, and rhodium prodrugs, of which the kinetics of release and in vitro and in vivo antitumor activity have been investigated against selected tumor cell lines, observing a 5 µM-higher activity with respect cisplatin with reduced systemic effects [134,135,136,137].

Three different polyphosphazenes containing tocopherol or testosterone glycinate and hydrophilic Jeffamine M1000 via the living cationic polymerization of Cl_3_P=NSiMe_3_ have been prepared and used to encapsulate and deliver camptothecin and epirubicin on MCF-7 cancer cells and MCF-7 spheroids. The hydrodynamic diameter of these nanoaggregates ranged from 142 to 253 nm, with the appropriate size to allow an extruded serum circulation with reduced renal clearance, showing similar or higher toxicity to MCF-7 human breast cancer cells as compared to the parent anticancer drugs, causing significant cell-cycle arrest in the G2/M phase and inducing significant apoptosis. Furthermore, camptothecin and epirubicin-loaded nanocarriers exhibited lower IC50 values than the parent anticancer drugs in MCF-7 spheroids [138].

**Figure 13:**
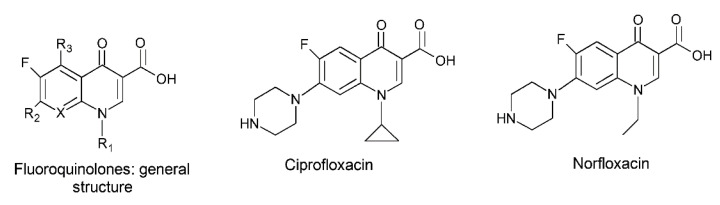
Some drugs easily encapsulated in polyphosphazenes bearing amino acid esters as side groups.

Polyphosphazenes containing the fluoroquinolone antibiotic substituents ciprofloxacin or norfloxacin (Figure 13) from 12 to 25 mol% and from about 88 to 75 mol% of amino acid esters, including alanine, glycine, and phenylalanine, have been prepared and the hydrolytic behavior has been studied, observing that it occurred at about a neutral environment. Antibacterial tests against *E. coli* showed activity as long as the antibiotic was released, thus suggesting the possibility to design devices for the controlled release of antibiotics [139].

Through the living cationic polymerization process, a series of multisubstituted POPs with controlled molecular weight and aqueous solubility bearing folic acid as tumor-targeting groups and hydrophobic anticancer molecules (through a pH labile linker) have been prepared. The polymers (tested at 25 °C and pH 7.4) showed to be stable over a short period of time in an aqueous environment but degraded over longer periods under simulated physiological conditions, thus demonstrating the potential of POPs to create tunable systems for the targeted delivery of anticancer drugs [140].

A series of chemically crosslinkable and thermoresponsive POPs as injectable biomaterial by using thiol, hydrophobic isoleucine ethyl ester, and hydrophilic amino-polyethylenglycol side groups, whose aqueous solutions at body temperature formed hydrogels suitable for administration by injection, have been prepared [141].

**Scheme 9:**
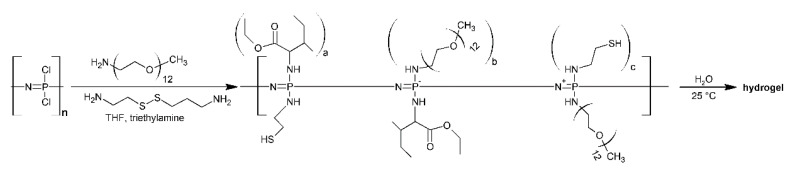
Injectable polyphosphazene–based hydrogel.

The copolymerization of hydrophobic systems such as polylactic acid and hydrophilic systems such as polyethylene oxide in thermosensitive poly(organophosphazene) hydrogel (based on hydrophobic isoleucine ethyl esters group and hydrophilic α-amino-ω-methoxy-PEG_550_) has been investigated for the delivery of hydrophobic drugs, such as doxorubicin and paclitaxel, even via intratumoral injection (Scheme 9) [142,143].

The hydrogel strategy has also been studied to achieve enzyme immobilization: a hydrogel based on methacrylate-substituted phosphazenes was demonstrated to immobilize lipase to an extent, depending on the hydrogel composition (maximum 24.02 mg/g); the immobilized enzyme activity decreased by about 50% only after four cycles of batch operation [144].

#### 3.1.3. Polyphosphazenes in Gene Therapy

Cationic POPs can, in principle, give rise to electrostatic interactions with anionic biomolecules such as DNA. Gene therapy involves the provision of cells with the required genetic information to produce specific proteins to modulate a given disease. Thus, the DNA must be delivered to the target cells and protected during derivation from metabolic processes. These results can be achieved through the preparation of cationic polymers which undergo noncovalent interactions with negatively charged plasmid DNA. In this frame, POPs bearing amines on P atoms have been proposed, observing a lower toxicity with respect the use of poly(2-dimethylaminoethyl)methacrylate [145]. POPs cosubstituted with 2-dimethylaminoethylamine and imidazole showed higher transfection activity compared to the corresponding systems without imidazole [146]. A series of poly[bis(2-(2-aminoethoxyethoxy)phosphazenes] have been investigated for their use in gene delivery [147], observing that the partial substitution of amine moieties with imidazoles improved the activity [148].

It has been reported that the presence of the polyphosphazenes was able to increase the efficacy/toxicity ratio over one order of magnitude, showing superior efficacies in a clinically relevant glioblastome primary cell-line (a synthetic strategy for the preparation of a library of polyphosphazenes of interest for gene delivery), thus establishing a new versatile, biodegradable polymeric gene delivery based on POPs with a high capacity for gene transfer efficacy in vitro and upon in situ treatment in vivo; for instance, forming polyelectrolyte nanoparticles by the coincubation of alkylamine and alkoxycarboxylate-POPs or by preparing water-soluble cationic POPs bearing alkylamine and imidazole groups [149,150,151]. The application of polyphosphazenes for gene delivery has remained relatively unexplored. A polyphosphazene platform, containing side-chain double-bond units to be reacted with alkanethiols, has been reported, which, combined with malic acids, were able to generate mixed polyelectrolyte complexes with a sufficient positive charge to bind polynucleotides and promote cell internalization but with the ability to destabilize cell membranes in response to pH. Then, systems have been elaborated as gene carriers to deliver nucleic acids as a potential means to treat glioblastoma, one of the most aggressive and malignant cancers (classified as class IV by the World Health Organization). A new strategy after surgical resection to prevent tumor relapse involves the delivery of either suicide genes or gene knockdown by siRNA directly to glioblastoma via intratumor administration. In vitro and in vivo evaluation has been carried out for gene delivery by using biodegradable poly [2-(2-aminoethoxyoxyethoxy)phosphazene] modified with lactobionic acid bearing a galactose group as a targeting ligand. Nanoparticles with a size around 130 nm have been achieved by condensing pDNA, which showed a higher transfection for BEL-7402 cells with lower cytotoxicity, with respect to the galactose-free systems and exhibited the selectivity of gene expression at a distant tumor site. Thus, the system could be a potential gene transfer vehicle for tumor targeting with low toxicity after intravenous administration [152].

Moreover, the potentiality of gene silencing mediated by siRNA has been explored for the treatment of genetic disorders and cancer where siRNA drugs can inhibit gene expression. Rapid enzymatic degradation in the blood of siRNA could be avoided by the use of hydrogel-based polyphosphazenes designed for the localized and long-term delivery of siRNA [153].

#### 3.1.4. Micelles, Liposomes, Polymersomes

Amphiphilic copolymers tend to self-assemble into a wide range of self-assembled nanostructures such as micelles and polymersomes in an aqueous environment due to different interactions of corresponding hydrophilic and hydrophobic groups. Polyphosphazenes have been studied either as carrier substrates by encapsulation using microspheres or micelles or by homogeneous dispersion of a drug in a biodegradable hydrogel or solid matrix. Liposomes were prepared by evaporating a chloroform solution of phosphatidylcholine and the phosphazenes polymer, treating with PEG and then hydration with HBS. Macrospheres, liposomes, and polymersomes have been prepared from amphiphilic ionizable polyphosphazenes by the incorporation of three critical moieties: polyethylene glycol octadecyl ether (C_18_(EO)_10_), aminobutyric acid (ABA), and ethylene oxide ethyl ether (EEE). The three units provide liposome-anchoring capabilities and are pH- and temperature-responsive, respectively. EEE was selected as having a lower critical solution temperature close to the physiological temperature (32 °C); ABA helps to modulate the critical solution temperature with respect to environmental pH and can confer biodegradability. These liposomes displayed pH-dependent release but were unstable under physiological temperature (37 °C) at a pH of 7.4 [154].

Of particular interest is the release of anticancer drugs. Many anticancer drugs currently used for chemotherapy are low-molecular-weight compounds (<1000 Da). They are administrated systematically orally or locally. Such molecules are known to have a short half-life (<2 h), a fast clearance in the blood circulation system, and attack not only tumor cells and tissues but, according to their level of selectivity, also normal cells, thus causing severe toxicity and side effects (nephrotoxicity, neurotoxicity, cardiotoxicity) which represent key dose-limiting factors in chemotherapy. Thus, different approaches to overcome such problems have been investigated and proposed. The strategies are continuously evolving based on new knowledge acquired on the physiological evolution of tumors.

Polyphosphazenes can either bind active tumor-targeting molecules (based on the affinity or reactivity of specific antigen/receptor overexpressed in the tumor cells or tumor tissues) and contemporary polyethylene glycol moieties to improve water solubility together with the drug, usually bonded through a stimuli-responsive spacer group.

The passive targeting strategies are some physical aspects of the interaction between the polymers and tumor cells. As an example, it was discovered that: (i) polymers with specific molecular weight can be preferentially accumulated in the solid tumor issues [155], (ii) macromolecules (i.e., nanoparticles) cannot permeate through the blood vessel pores of normal tissues with a regular structure, and (iii) it is difficult for polymer particles which have entered in the tumor tissue to be drained off, as they are not present in the lymphatic vessel [156].

In this frame, polyphosphazenes represent an excellent resource to develop newly designed drug carriers for tumor targeting, tailored to meet various requirements such as water solubility, chemical stability, biodegradability, compatibility with the drug, and targeting properties forming micelles or microspheres, which, upon diffusion and degradation, can release the targeted drug [15]. One-pot synthesis of crosslinked POPs dopamine microspheres for controlled drug delivery has been reported by reacting in acetonitrile HCCP, triethylamine, and dopamine at 50 °C for 3 h under ultrasonic irradiation (53 kHz, 150 W). Cyclomatrix polyphosphazene microspheres have been achieved which are able to absorb acriflavine (19.5 mg acriflavine/gram of microsphere), as a model drug, and which release the drug for a long time depending on the pH (29% released in acidic medium; 47% at neutral pH) for up to 7 days [157]. A new class of tripodal amphiphiles for self-assembly to bilayered polymersomes, based on cyclotriphosphazenes bearing equimolar amounts of hydrophilic polyethylene glycol and a hydrophobic oligopeptide, have been proposed, for which the shape (micelles or polymersomes) resulted depended on the hydrophobicity of the oligopeptide [158]. The reaction of HCCP and 4,4′-sulphonyldiphenol in the presence of triethylamine in acetone at 30 °C in an ultrasonic bath (100 W, 80 kHz) for 4 h gave hollow microspheres via a self-assembly process [159].

**Scheme 10:**
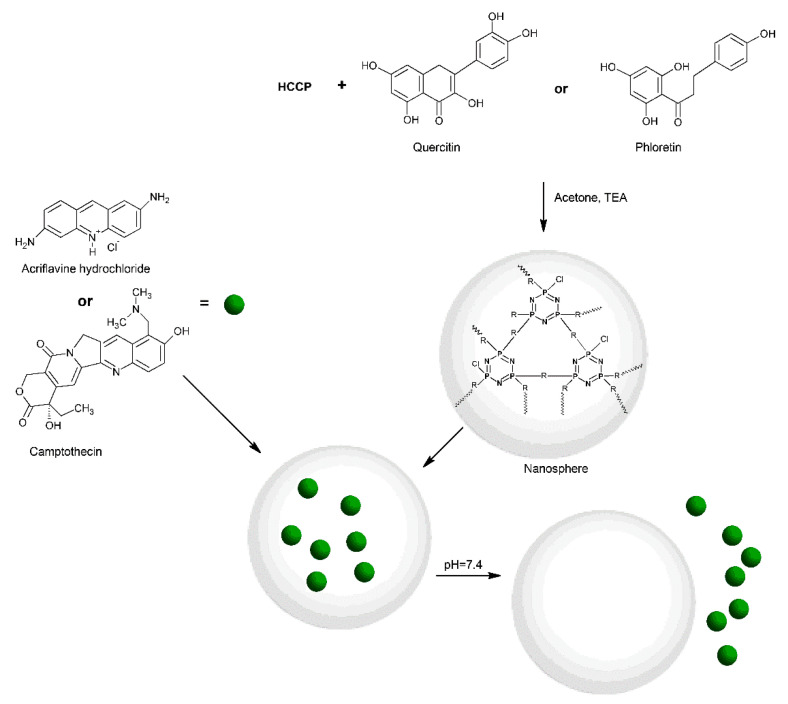
The pH–responsive nanospheres and microspheres for drug delivery.

Cyclomatrix polymers with quercetin as bridging moieties have been used to prepare nanospheres to study the release of acriflavine as a model drug, which can be stored in the nanosphere at 37 °C up to 41% and released in 11 days at a pH of 7.4. The hydrogen-bonding interaction between acriflavine molecules and quercetin nanospheres may also contribute to the steady release rate (Scheme 10) [160]. Radical-containing microspheres based on a cyclophosphazene core and phloretin polymeric arms have been prepared by reaction with (2,2,6,6-Tetramethylpiperidin-1-yl)oxyl (TEMPO) and used as drug loading (camptothecin) while investigating the drug release at different pH levels: it was reported that 41% of camptothecin was released at a pH of 4.0 and 32.6% at a pH of 7.4 from microspheres after 350 h, respectively (Scheme 10) [161].

**Figure 14:**
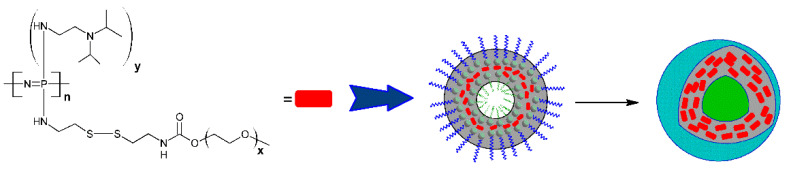
Polyphosphazene-based polymersomes.

A related strategy has been used to prepare biodegradable and antioxidant phosphazene tannic acid nanospheres [162]. Polymersomes present unique structural architectures with an interior aqueous core surrounded by a typical bilayer membrane formed by the association of hydrophobic parts. The bilayer membrane is the characteristic of polymersomes, which allows to separate the inside and outside aqueous environments with different compositions and concentrations based on the selective permeability of the membrane. A wide variety of polymersomes have been developed and their stimuli-responsive properties have been investigated, thus allowing drug release. The hydrodynamic diameter ranges between 150 and 250 nm. Cancer cells have reductive and acidic environments as compared to normal body cells, thus reductive/acidic-responsive polymersomes may play a crucial role in cancer therapy to release the loaded drug. Three different reductive/acidic-responsive polyphosphazene bearing mPEG-SS-amino and N,N-diisopropylethylenediamine arms in different amounts have been prepared which self-assembled in polymersomes. Hydrophilic/hydrophobic drugs (Doxorubicin/HCl and Doxorubicin) have been encapsulated into polymersomes with high-loading and high-encapsulation efficacy due to the strong intermolecular interaction. The drug release rates were observed to depend on the acidity/reductive properties of the medium (Figure 14) [163].

Colic acid has a high-binding affinity to the foresaid X receptor (FXR, which is overexpressed in most of the cancer cells), thus colic acid has been grafted to poly(bis-carboxyphenoxy phosphazene)poly diallyl dimethylammonium chloride to prepare nanomicelles with a hydrodynamic diameter of around 218 nm. Colic-acid-conjugated hybrid polymeric micelles targeted the FXR with paclitaxel loading have been shown to improve the therapeutic efficiency without systemic toxicity [164,165]. Thermoresponsive nanoparticles based on poly[bis(carboxyphenoxy)phosphazene]-polylactic acid polymers demonstrated the capacity to encapsulate the hydrophobic drug paclitaxel with a pH-dependent release capability due to the pH-responsive quenching of the polymers. [151] The kinetics of the encapsulated probe release of 8-hydroxypyrene-1,3,6-trisulfonic acid has been studied to improve the lifetime as can be achieved by introducing PEGilated chains acting as sterical barriers between opsonin and other siero-proteins [166].

Cyclophosphazenes bearing and oligopeptide arms of the type [NP(mPEG_350_)(GlyPheLeuAspEt_2_)]_3_ have been used to prepare very stable micelles due to their ability to self-assemble, where the hydrophobic blocks of the copolymers form the core of the micelle and the hydrophobic block the coronas or outer shell of the micelle [167]. These micelles, used as new drug systems, offer many advantages: they have a very low critical micelle concentration (about 0.1 mg/L), are thermoresponsive, biodegradable, and allow high solubilization of hydrophobic drugs. Furthermore, the trimer backbone is monodispersed, thus showing control of the molecular weight, and the variety of functionalization allows a design for specific drugs. Some preliminary intriguing results have been reported [168].

**Figure 15:**
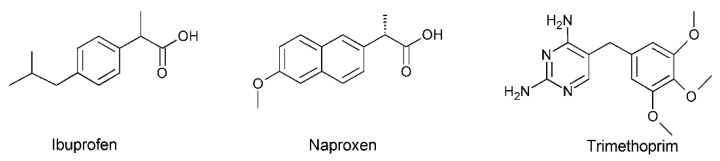
Some drugs encapsulated in polyphosphazene-based microspheres.

Microspheres based on cyclophosphazenes have also been proposed as ibuprofen [169] and antibiotic (trimethoprime) carriers and their controlled release (Figure 15) [170].

Microspheres for application in periodontal disease and implant surgery have been prepared by dissolving succinylsulfathiazole or naproxen and a polyphosphazene-bearing phenylalanine ethyl ester and imidazole as side groups. In vivo release studies and surgical trials (on male rabbits) have been carried out observing no signs of inflammation, but no reparative bone or osteoid tissue was found [81].

#### 3.1.5. Nanoparticles

**Figure 16:**
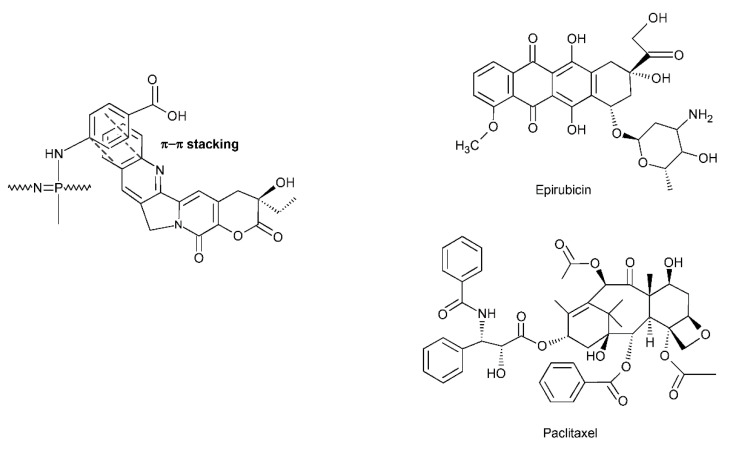
The π–π stacking between the polyphosphazene and camptothecin. Epirubicin and paclitaxel can give similar π–π stacking with the polymeric chain.

An intriguing aspect of this biochemistry is represented by the hydrodynamic diameters of the nanoparticles: if higher than 200 nm, they are considerably bigger than the renal filtration clearance limit (about 5.5 nm), thus leading to a long circulation time in the bloodstream; if they are of smaller size than 400 nm, their possible retention in the vascular regions after intravenous administration in close contact to tumor sites can be achieved [155]. Nanoparticles (NPs) of poly[(ethylamino benzoate)(ethylglycinate)]phosphazene have been prepared and loaded with camptothecin: the in vitro drug release behaviors were studied at a pH of 7.4 and 5.6. The ability of the nanoparticles to interact with the hydrophobic drug has been explained by a π–π interaction between the aromatic ring of camptothecin and the polymer, modulating the drug loading and release depending on the benzoate amount along the polymeric chain (Figure 16) [155].

Nanohybrid systems based on gold-poly(carboxyphenoxy)phosphazene have been prepared and investigated as a stimuli-responsive drug delivery of AuNPs: it was observed that the drug loss is low at a neutral pH, whereas rapid drug release was noticed after the internalization of nanoparticles by the cancer cells. Recently, the designed preparation of polymer-functionalized AuNPs has attracted increasing interest either for improving the stability of NPs or to tailor the chemical/physical/surface properties of NPs [108,171].

**Figure 17:**
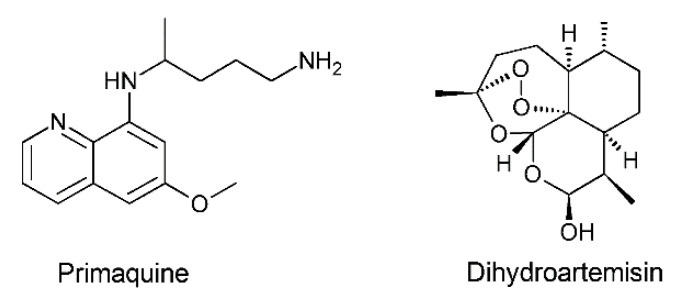
The two antimalarial drugs tested in polyphosphazene nanoparticles.

Poly[bis(carboxy phenoxy)phosphazenes] nanohybrid systems exhibited excellent dispersity and stability, reducing the loss of drugs in normal tissues with an efficient internalization of AuNPs into tested cells (MDA-MB-231) with a strong cytotoxic effect through the induction of apoptosis. Moreover, pH- and thermoresponsive-NPs composed via choli acid poly(biscarboxyphenyl)phosphazene-polylactic acid have been reported which showed reversible gelation behavior in the temperature range 20–37 °C and a drug-release capability at an acidic pH due to the pH-responsive quenching effect of the hybrid polymer. The release of paclitaxel was observed over 12 days. It is noted that the drug release from the NPs was effectively controlled by the mechanical strength of the polymer [165]. Multilayered NPs have been prepared by poly[di(sodium carboxyphenoxy)phosphazene] and poly(diallyldimethyl ammonium chloride) deposited on the CaCO_3_ nanoparticles’ surface of a diameter of 237 nm, exhibiting a high-drug-loading content with enhanced cellular uptake. Under acidic conditions, the multilayer structure controls burst release, providing sustained drug release for a long period. Chrysin (an angiogenesis-inhibitor-activating ROS species) and cisplatin have been incorporated and have been tested against oral carcinoma cells, observing a 92% regression volume as compared to cisplatin alone loaded in the same nanoparticle. The work provided a simple method to formulate multiple drugs in single nanosystems [172]. Nanocarriers with sizes ranging from 200 to 240 nm have been prepared by dissolving POPs substituted with 2-propoxy, 4-acetamidophenoxy, 4-formylphenoxy, or 4-ethoxycarbonylanilino arms in dichloromethane together with the antimalarial drugs primaquine and Dihydroartemisinin (Figure 17) by emulsioning in the presence of a surfactant under mechanical stirring. In vivo (mice) antimalarial efficacy was tested: it was shown that nanoparticle formulations were effective in eradicating completely the parasites after 14 days, but at a lower dose than standard drug combinations [173].

#### 3.1.6. Nanofibers

Electrospinning methodology has been reported to prepare nanofibers, membranes, and scaffolds in view of different applications [36,174]. A core sheath nanofiber membrane with poly[bis(p-methylphenoxy)phosphazene] and polyacrylonitrile has been prepared and studied for enzyme (lipase) immobilization [175]. Coelectrospun composite nanofibers (with a diameter ranging from 240 to 430 nm) of blends of poly[(amino acid ester)phosphazene] (alanino ethyl and glycinoethyl) and gelatin have been studied as scaffolds for cells adhesion and growth [176]. Eletrospun fibers have been prepared using polyphosphazene bearing *l*-proline methyl ester and 4-hydroxy-*l*-proline methyl ester as side arms to achieve a new bioactive material for bone repair. The biomimetic mineralization was tested on the fibers and on the bulk polymer, observing in both cases bioactivity with the formation of an abundant calcium phosphate layer after 24 h and the adhesion of calcium phosphate crystals to the fiber mimicking the hydroxyapatite growth in collagen fibers [177]. Poly[(ethyl alanato)(p-methyl-phenoxy)phosphazene] has been used to modify the surface of the electrospun fibers of poly(ε-caprolactame) for tendon tissue engineering, to improve the hydrophobicity of the matrix, and to enhance the protein synthesis by seeded Human Mesenchymal Cells (hMSCs). The work demonstrated the enhanced cellular response with cell adhesion and long-term cell infiltration through the matrices with the phosphazene-modified surface [178].

### 3.2. Phosphazenes as Immunoadjuvants

Some reviews on polymeric genomics stimulated the investigation on the role of polymers in the induction of specifically genetically controlled responses to antigens, focusing on the cooperative interactions of polymers with plasma cell membranes and the trafficking of polymers to intracellular organelles [179]. In this frame, POPs can exploit a significant role to investigate a possible structure–activity relationship with the aim to design suitable controlled supramolecular assemblies, forming nanospheres or microspheres [15,180]. Thanks to chemical versatility, POPs have been proposed as immune adjuvants, having a flexible backbone, hydrophobic spacers, a high molecule weight, and a partially dissociated ionic group of molecules able to form water-soluble complexes with many biological targets, including proteins, which are essential for their immunostimulating activity through a long-lasting immune response with high-antibody titers [181,182]. The immune adjuvant polymers must be water soluble and usually contain carboxylic acid groups. Poly[di(carboxylatophenoxy)phosphazene] can form noncovalent interactions with protein antigens and demonstrate activity in animal and human clinical trials. Examples are the complexation with group-specific antigen (Gag antigen), for which the presence of the polymer induced the maintenance of the immunostimulation and facilitated the effective delivery of the antigen to the target cells [183]. Combination with H5N1 influenza vaccine, of which thermal stability resulted enhanced in solution, provided for a dose-sparing effect in vivo [184].

Polyphosphazene adjuvant technology evolves through the discovery of new and more potent derivatives and the investigation of alternative delivery routes, such as mucosal and intradermal. It was reported that formulations based on poly[(sodium carboxylate ethyl phenoxy)phosphazene] containing different doses of the influenza X-31 antigen or bovine serum albumin were shown in mice to enhance the antibody responses up to 1000-fold. Even if the detailed immunological mechanism deserves further investigation, some empirical evidence has shown to have a relevant role: the molecular weight of POPs linked to complex stability, the degree of complex compaction linked to antigen presentation and antigen loading, together with the ability of the antigen–POP complexes to adsorb on the cell surface, stimulating intracellular ionic fluxes. Some results of clinical trials have been published. A phase I clinical study on the A/Johannesburg/33/94(H3N2) strain with a 500 µg dose of poly[di(carboxylatophenoxy)phosphazene] showed to be very efficient. The adjuvanted vaccine produced a 14.7-fold increase in antibody titers compared to a 3.1-fold increase for the nonadjuvanted one, with no serious adverse events [185]. The role of the POPs was also investigated in clinical trials with 100 mg of oligomeric HW-1 Gp 160 mm/LAI-2-vaccine in HIV-seronegative volunteers, observing a higher immunization without serious adverse vaccine-related events [186].

**Scheme 11:**
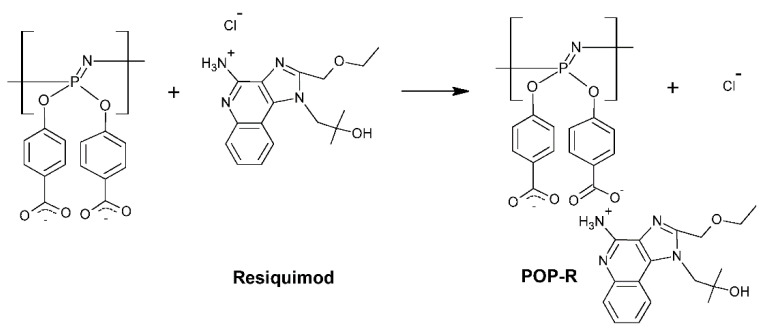
The ionic interaction between the water soluble polyacid–polyphosphazene and Resiquimod.

Intradermal administration of the vaccine is an intriguing objective, as the skin acts as a natural barrier against infections and has a high density of dendritic cells (such as Langerhans cells) whose formation is to recognize foreign pathogens. To overcome the stratum corneum barrier and increase skin permeability, different approaches have been explored:

(i) The use of microneedles, submillimeter structures capable of penetrating the stratum corneum and releasing the vaccine in the appropriate skin compartment: hollow microneedles which allow infusion of liquids formulation into the skin or microneedles with solid state vaccine formulation [15,187];
(ii) Nanoscale constructs [188], as in the case of the spontaneous self-assembly of Resiquimod with a water-soluble poly[di(carboxylatophenoxy)phosphazene] forming an ionically paired system and a ternary one, including the Hepatitis C virus antigen. It was demonstrated that the supramolecular assembly enabled high immunostimulation in cellular assays (mouse macrophage reporter cell line) and in vitro hemocompatibility (human erythrocytes). Moreover, in vivo studies gave quite promising results (Scheme 11) [189].

The adjuvant platform based on water-soluble poly[di(sodiumcarboxylatoethylphenoxy)phosphazene] for the needle-free intradermal subunit vaccine, the Bovine Viral Diarrhea Virus (BVDV) type-2 E2 protein/TriAdj against bovine viral diarrhea virus, which is one of the most serious pathogens in cattle, has been developed. It was observed that the intradermal vaccine induced robust humoral and cell-mediated immune responses equivalent to the 1M delivery, indicating that the intradermal route is very suitable and practical for vaccination in cattle, being less painful and with the possibility to reduce the antigen dose [190]. A similar strategy has been proposed for intradermal immunization with inactivated Suine Influenza Virus (SIV) H1N1 coadministrated with poly[di(sodium carboxylatoethylphenoxy)phosphazene]. A stimulated significant anti-SIV antibody titer, an increment of neutralizing antibodies, and a significant reduction of lung virus load with the limited reduction of gross lung lesions after a challenge with virulent SIV-H1N1 relative to control animals was observed [191,192].

Cancer immunotherapy is one of the most attractive innovative approaches, having identified some specific tumor antigens. TSA/TAA soluble proteins must be presented by antigen-presenting cells (APCs) but are generally weak in immunogenicity, thus requiring a delivery vehicle that can improve cellular uptake, reducing the elimination from circulation. Various nanosystems have been used for protein delivery, such as liposomes, nanogels, micelles, and solid nanoparticles, even if some problems still remain in their application, such as poor loading due to high water solubility and the big bulk size of proteins. Polymersomes containing an aqueous interior could offer protein high-loading but it is more stable than liposomes: polymers responsive to various stimulations (pH, temperature, redox conditions, light) must be used to form polymersomes. Once these stimuli have been applied, they will provide the disintegration of polymersomes or the “breathing” vesicles with enhanced permeability to release small drugs, even if they have been rarely reported for antigen delivery [193,194,195]. POPs with N,N-diisopropylethylene diamine as hydrophobic side groups and water-soluble polyethylene glycol have been used to achieve polymersomes to deliver ovalbumin, a model antigen for immunological studies [196].

Human respiratory syncytial virus (RSV) and parainfluenza virus type 3 (PIV3) are major causes of serious lower respiratory tract disease in infants: currently, no licensed vaccines against RSV and PIV3 are known. Mice, cotton rats, and hamsters were immunized intramuscularly with a formulated chimeric glycoprotein based on poly[di(sodium carboxylatoethoxyphenoxy)phosphazene], thus representing a safe, effective, potential bivalent vaccine against both RSV and PIV3 [197].

**Scheme 12:**
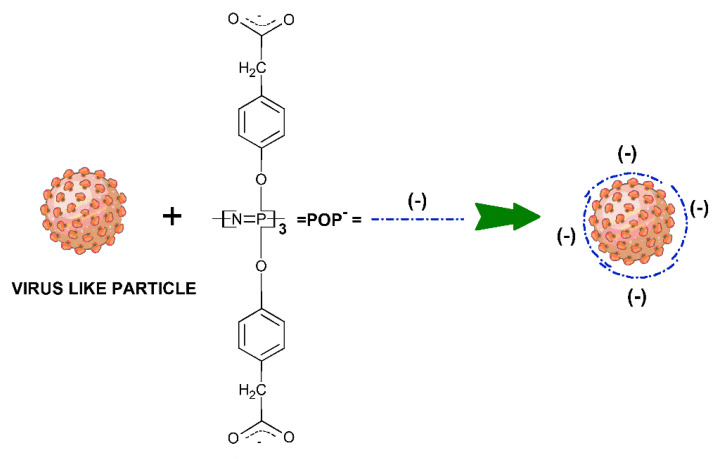
Virus-like particles in polyacid–polyphosphazene. (Virus image by brgfx on Freepik).

Recently, poly[di(carboxylatomethylphenoxy)phosphazene] and poly[di(carboxylatoethylphenoxy)phosphazene] have been proposed as immunoadjuvants for in vivo experiments with human papillomavirus-like particles based on the RG1-VLPs vaccine. Stabilization of the antigenic particles was observed and immunization in mice demonstrated increased immune responses (Scheme 12) [198,199].

**Figure 18:**
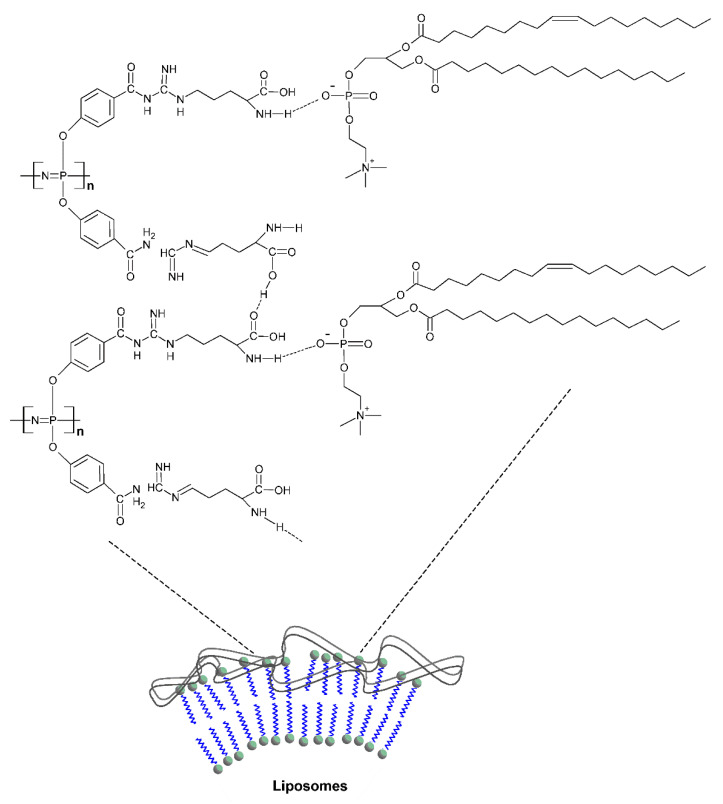
The polyphosphazene–arginine–layered liposome.

The multifunctionality of polyphosphazenes has also been exploited in the preparation of layered liposomes based on poly[di(carboxyphenoxy)phosphazene] functionalized with arginine able to encapsulate rifampicin and isoniazid drugs against tuberculosis, observing a controlled intracellular release and an immunomodulation effect with the activation of macrophages (Figure 18) [200].

### 3.3. Phosphazenes in Tissue Engineering

One of the most intriguing applications of polyphosphazenes materials is represented by tissue engineering as scaffolding materials. Materials resembling natural bone must display inductive effects in stimulating bone repair. Biodegradable polyphosphazenes have demonstrated advantages over polyesters in inducing bone regeneration due to the PN backbone and the organic side groups designed to confer the physicochemical and biological properties of the resulting materials. It was shown that the osteogenic differentiation of osteoblasts and bone mesenchymal stromal cells is significantly enhanced on polyphosphazenes both in vivo and in vitro in comparison with biodegradable polyesters. Polyphosphazenes with hydrolytically labile side groups (i.e., glycolyl, glycerol, imidazolyl, glycolide, and amino acids) can be used as biomaterials in both tissue engineering and drug delivery, with degradation rates dependent on the combination of the side groups [201]. Amino-acid-ester-substituted polyphosphazenes can hydrolyze into nontoxic compounds such as amino acids, phosphate, and ammonium ions. Polyphosphazenes bearing smaller amino acids such as glycine or alanine are mechanically soft and fast degrading, while bulkier groups such as lysine result in slow degrading. Cosubstituted polyphosphazenes were developed to achieve polymers with designed and tunable mechanical and degradation properties [88]. The additional properties exhibited by these materials, such as the glass transition temperature in a large range (from −40 to +42 °C), the tensile modulus from 30 to 450 GPa, and the lower surface energy, render polyphosphazenes versatile materials for tissue engineering applications. Furthermore, biodegradable polyphosphazenes have been processed into different porous scaffolds via salt leaching, microsphere sintering, and electrospinning, or blended with other biodegradable polymers such as poly(lactic acid- gluconic acid) [202].

#### 3.3.1. Bone Tissue Engineering

Polyphosphazenes are good choices for the objective of developing multifunctional materials with antibacterial and antioxidant activity, electroactivity, and osteoinductivity for the efficient regeneration of infected bone defects, owing to the chemical flexibility and biodegradable alternate phosphorus and nitrogen atoms backbone, which can be easily functionalized with amino acid esters, giving rise to biocompatible materials for in vivo implementation with the inherent capacity to provide osteogenesis. Among all the biodegradable polymers developed for bone regeneration, polyphosphazene is especially worth mentioning, as it is easily modified and tailored to the physicochemical properties of bone regeneration.

**Figure 19:**
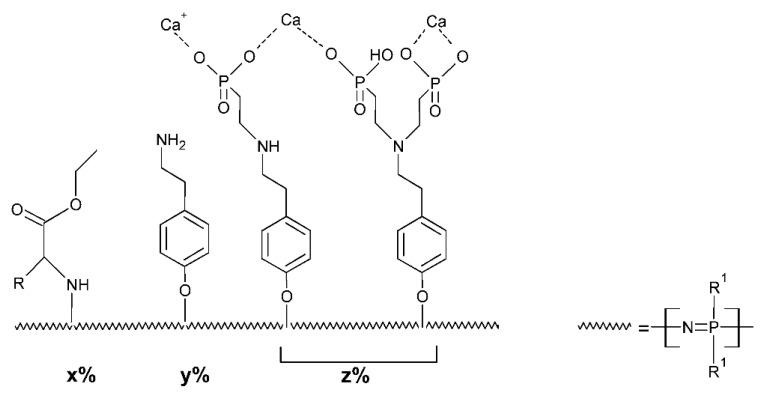
Polyphosphazene–calcium phosphate composites.

Bone tissue consists of mineral, collagen, and noncollagenous proteins, where hydroxyapatite [Ca_10_(PO_4_)_6_(OH)_2_] represents about 70% by weight. Phosphazene–hydroxyapatite composites (Figure 19) have been produced via a reaction of calcium phosphates with poly[(ethyloxybenzoate)phosphazene], poly[(propyloxybenzoate)]phosphazene, and poly[bis(sodium or potassium carboxylatophenoxy)phosphazene] [203,204,205], whose chemical, physical, and morphological properties have been investigated to match the structure of bone and to propose these materials as bone cement. Polyphosphazene/nano-hydroxyapatite composite microspheres have been reported which showed good osteoblast cell adhesion.

**Figure 20:**
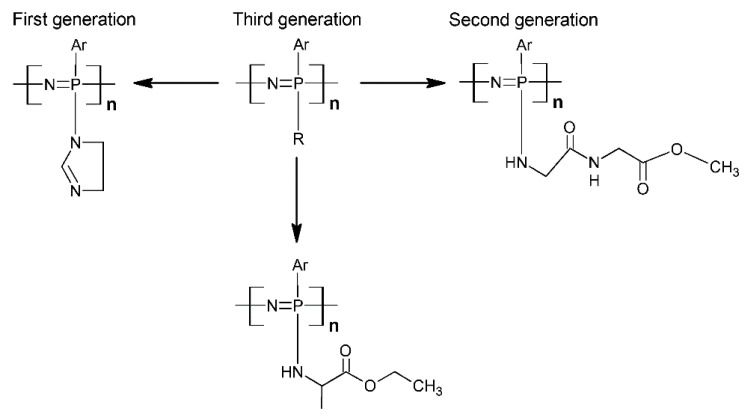
Evolution of design of polyphosphazenes for bone regeneration.

Different strategies have been explored within the time to achieve the most suitable polyphosphazene system for bone regeneration (Figure 20).

**Figure 21:**
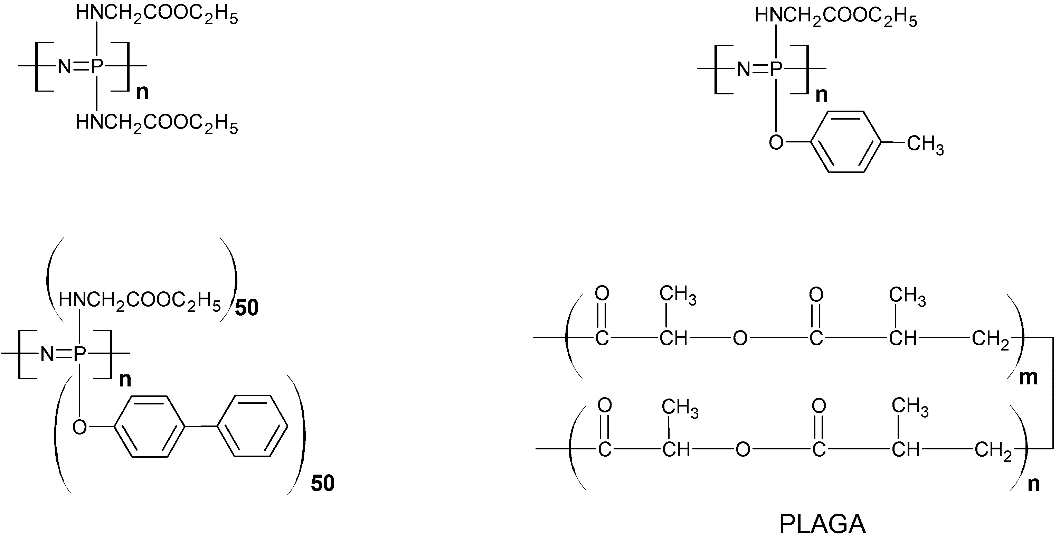
Biodegradable glycine containing polyphosphazenes for bone regeneration.

**Figure 22:**
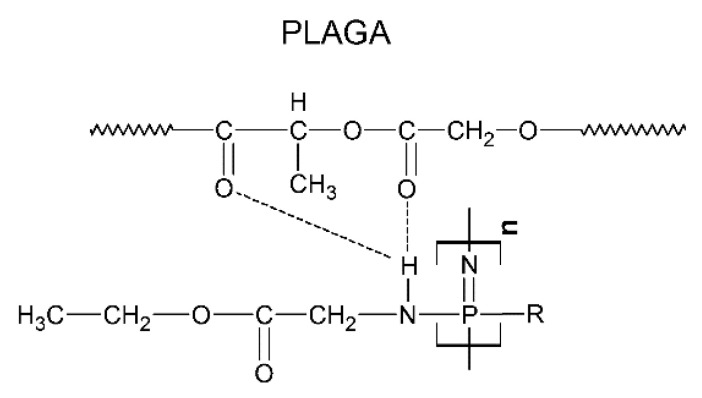
H-bonds between polymeric chains in polyphosphazene–PLAGA blends.

The first generation of biodegradable polyphosphazenes was designed with an imidazole side group (a biocompatible group able to confer hydrolytic instability to the backbone and nontoxic degradation products): they showed significant enhancement in alkaline phosphate activity when compared to poly(lactic-acid-co-glycolic-acid)(PLAGA), but a decrease of cell attachment and growth with the increase in the content of imidazolyl groups [206]. Histological studies demonstrated that poly[(50% p-methylphenoxy)-(50% ethylglycinato)phosphazene] and poly[bis(ethylglycinato)phosphazene] appeared to support bone growth to a comparable extent to the control PLAGA [207]. The second generation amino-acid-ester-containing polyphosphazenes were developed to achieve a higher biocompatibility. An increase in the content of the ethyl glycinate groups favored increased cell attachment and growth, with a controlled degradation rate depending on the hydrophobic and steric hindrance side groups [208]. Some inflammatory responses for the PLAGA materials used for bone regeneration and the unexpected structure failure have been observed and partially solved by blends with polyphosphazenes. Poly[(glycineethylester-co-alanine ethyl)phosphazene] gave honeycomb-patterned films with enhanced protein adsorption and apatite deposition in simulated body fluid and showed great advantages in promoting osteogeneous differentiation [209]. It was demonstrated that the nature and the ratio of the pendent groups bonded to the P=N backbone play a relevant role in determining the mechanical properties of the resulting polymers and the cell adhesion (Figure 21) [210]. The third generation of dipeptide-substituted polyphosphazenes was developed to achieve more miscible blends with poly(lactic acid-glycolic acid), PLAGA, by substituting the ethylglycinate side groups with glycylglycine ethyl ester side groups, thus achieving PLAGA blends with intermolecular H-bonds. It was observed that this material was self-assembled into interconnected microspheres (Figure 22) [211].

**Scheme 13:**
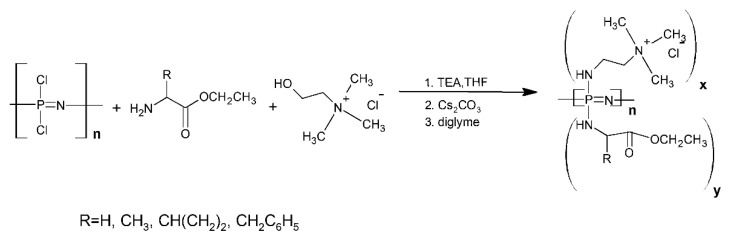
Synthesis of poly[(aminoethylester)_x_(choline chloride)_y_–phosphazene].

To improve the osteoblast activity, choline chloride and glycine, alanine, valine, and phenylalanine ethyl ester were bonded to the P=N backbone in cyclic trimers and polymer phosphazenes were blended with PLAGA, achieving materials with osteoblast proliferation with high osteoblast phenotype expression (Figure 22 and Scheme 13) [212,213]. Injectable hydrogels based on polyphosphazenes able to promote osteogenesis were also prepared for a bone regeneration effect by bone morphogenetic protein-2 release [214] and systems bearing *l*-isoleucineethylesters, α-amino-ω-methoxy PEG_750_, and 4-(2-aminoethoxy)4-oxobutanoic acid as side-chains were tested on three young male beagle dogs with mandibular defects to induce bone augmentation in the alveolar bone for the successful placement of dental implants. Twelve weeks after the treatment, significant bone generation occurred with high-osseointegration levels [215].

**Scheme 14:**
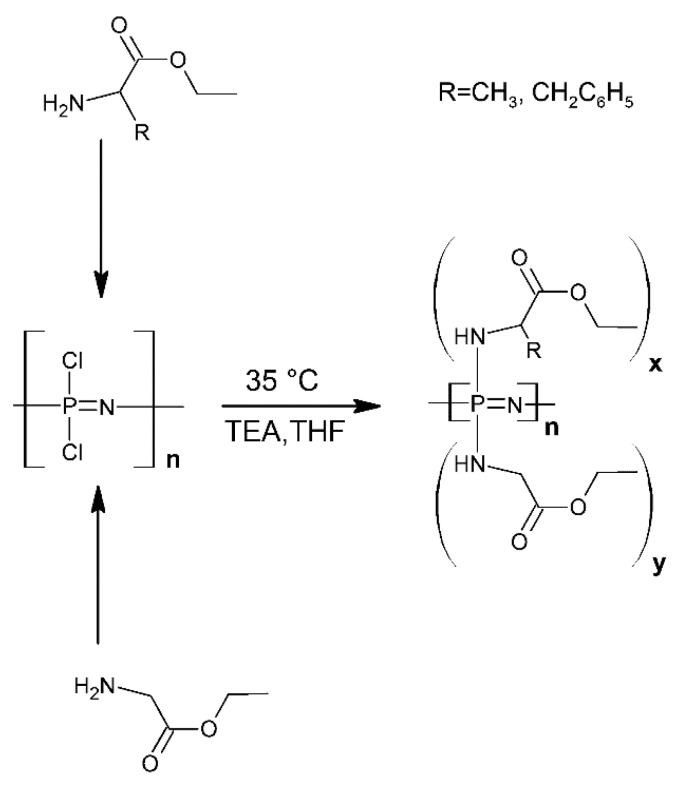
Synthesis of poly(ethylalanate)_0.3_(ethylglycinate)_0.7_]phosphazenes and poly[(ethylphenylalanate)_0.3_(ethylglycinate)_0.7_]phosphazenes.

A key point to understand the role of phosphazenes in bone tissue engineering is the mechanism of stimulation of osteogenesis and osteogenic differentiation. Comparative cell (mesenchymal stromal cells) culture experiments were performed by culturing on poly(ethylalanate)_0.3_(ethylglycinate)_0.7_]phosphazenes and poly[(ethylphenylalanate)_0.3_(ethylglycinate)_0.7_]phosphazenes by adding quantitative inorganic phosphate as polyphosphazene degradation products into trans good chambers. The results revealed that both the films and the degradation products play a significant role in regulating cell behaviors, with poly[phenylglycinate]phosphazene (PPGP) films able to give great promotion in osteogenic differentiation via the contact effect [216], likely due to the slower degradation rate (Scheme 14).

**Scheme 15:**
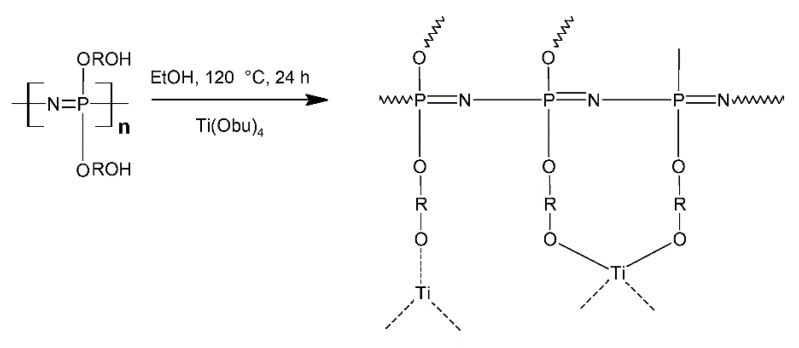
Synthesis of a Ti(OBu^t^)_4_–phosphazene composite.

A further aspect to consider for designing bone tissue engineering materials is to imitate the composition, morphology, and physiological characteristics of natural bone tissue, including sensitivity to electrical stimulation, originated by the structural arrangement of collagen fibers and hydroxyapatite nanocrystals. This feature suggested the use of electrical stimulation to accelerate bone regeneration and some intriguing and promising results upon seeding mesenchymal stromal cells on polymeric conductive substrates on polypyrrole and polyaniline [217,218,219] and in conductive composites based on polylactic acid incorporating carbon nanotubes [220]. Carbon nanotubes (CNT) have also been dispersed in solutions of alanine ethyl ester and glycine ethyl ester cosubstituted polyorganophosphazenes, thus preparing conductive composite films on which assays on cell attachment, proliferation, and differentiation were conducted. It was observed that appropriate electrostimulation (1.5 V, 2 h per day) improved the increment in the expression of osteogenic markers as alkaline phosphatase. Collagen I and calcium deposition occurred, likely due to the higher amounts of ions attracted together with the activation of voltage-gated Ca^2+^ channels on cell membranes, thus increasing the level of intracellular Ca^2+^ and thus promoting osteogenesis [221]. A biocompatible composite able to induce cell proliferation and osteoblastic differentiation has been achieved by the hydrothermal crosslinking of water-soluble phosphazene containing hydroxy groups and Ti(OBu)_4_, as seen in Scheme 15 [222].

New porous scaffolds based on polyphosphazene bearing dimethyalminoethane/calcium phosphate containing chitosan microspheres showed a very good osteogenic potential of cells, thus suggesting that they can be successfully utilized in bone tissue engineering [223]. Scaffold materials based on electrospun polydopamine-modified polyphosphazene have been reported to exhibit a higher osteocompatibility than aliphatic polyesters, with significant enhancement in MC3T3-E1 cell attachment and proliferation [224].

Also in vivo, tests have been carried out by using glycylglycine ethyl-ester-substituted polyphosphazene and poly(lactic-co-glycolic acid)blends in a rabbit critical-sized bone defect model. Based on radiological and histological analyses, bone regeneration and a mild inflammatory response were observed, proving these materials to be viable for matrix-based bone regenerative engineering [225].

A recent improvement in this topic is the preparation of AgNPs loaded with poly[(aniline tetramer)(ethyl glycyl)]phosphazene, followed by polydopamine (PDA) modification, forming PATGP@PDA+Ag microspheres, which demonstrated strong antibacterial activity against *S. aureus* and the most abundant neobone formation after coimplantation of these microspheres with *S. aureus* into rat calvarian defects. The data revealed that AgNP-loaded scaffolds made of conductive polyphosphazene were promising for the regeneration of infected bone defects [226].

#### 3.3.2. Polyphosphazenes in Nerve and Cardiac Tissue Engineering

The ability of polyphosphazenes to yield materials able to promote cell adhesion, proliferation, and differentiation, depending on the nature of the substituents bonded to the P=N backbone, allowed us to design systems suitable for restoring or replacing damaged tissues [227]. Electrospun nanofibers (0.1–2.3 µm diameter) based on poly[(ethyl phenylalanato)_1.4_(ethylglycinato)_0.6_phosphazene] have been prepared and characterized. The degree of the endothelial cell proliferation after 4 days on the scaffolds prepared with these fibers was higher than that on the polystyrene tissue-culture plates [228]. Three-dimensional porous scaffolds for tissue regeneration with tuneable degradability and morphology have been also prepared through the photopolymerization of glycine-substituted polyphosphazenes bearing thiol moieties. Adipose-derived stem cells, with high potential for tissue engineering, have been successfully tested both for adhesion and proliferation [229].

Electrospun polycaprolactone nanofiber (400–4000 nm diameter) matrices functionalized with poly[(ethyl alanato(p-methyl phenoxy)phosphazene] have been reported to improve adhesion, proliferation, and differentiation of osteogenic and chondrogenic cell lines BMP-2 and BMP-7, respectively, in a higher extent with respect nonfunctionalized polycaprolactone, thus being promising materials for tendon/tear repair [230]. Amino-acid-ester-substituted POPs have been studied and considered good candidates for ligament and tendon engineering due to the tendency to form films, a tuneable hydrolysis rate, and designed mechanical properties depending on the steric hindrance of amino acid esters and the presence of UV-curable citronellol [231].

Tubular matrices of poly[bis(ethylalanato)phosphazene] have been tested as guides for nerve regeneration [232]. The insertion on the P=N backbone of electroactive moieties suggested the possibility to design suitable POPs for nerve tissue engineering [233].

The fibers of poly[bis(ethylalanato)] and poly[(ethylphenylalanato)_0.8_(ethylalanato)_0.8_(ethyl glycinato)_0.4_phosphazene] have been shown to possess high cell adhesion and proliferation: their capacity to improve rat neuromicrovascular endothelial cell growth has been tested [234].

Fluoroalkoxy-substituted polyphosphazenes have been prepared and their elastomeric, hydrophobic, and antimicrobial properties have been explored for a possible application in cardiac tissue engineering [235].

### 3.4. Other Biomedical Applications

**Scheme 16:**
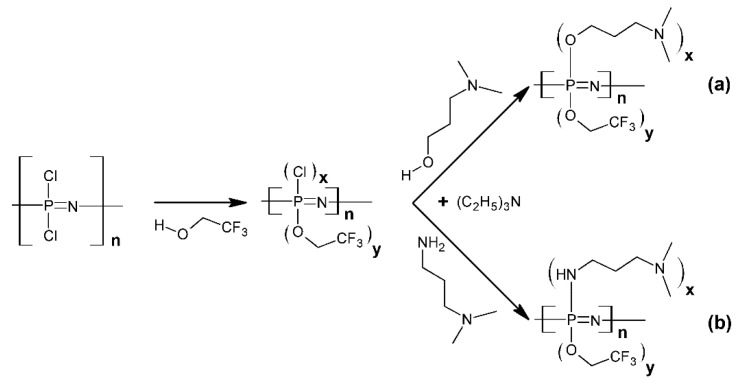
Synthesis of hydrophobic trifluoroethoxy-phosphazenes bearing tertiary amino groups linked to the phosphazenes backbone (**a**) via oxy bridge and (**b**) via amino bridge.

Polyphosphazenes bearing octafluoropentoxy chains blended with polyurethane or crosslinked gave textured films which showed the inhibition of adhesion and biofilm formation [236], thus representing a good biomaterial to prevent pathogenic infections and thrombosis in the application of blood-contacting medicinal devices [237,238,239]. Poly[bis(trifluoroethoxy)phosphazene nanocoated-stainless-steel stents were implanted in the renal and iliac arteries of minipigs. Reduced stent stenosis and lower inflammation response have been observed (Scheme 16) [240].

**Scheme 17:**
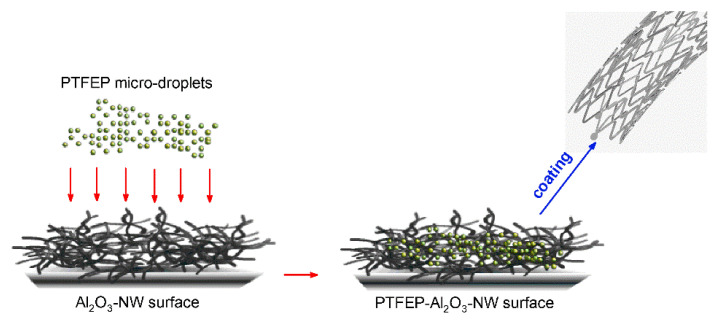
Schematic description for preparation of hydrophobic fluorinated-phosphazene-aluminum-oxide nanowires as coatings for cardiovascular stents. (Stent picture from https://free3d.com/3d-model/medical-stent-5591.html, accessed on 20 October 2022).

Nontoxic, superhydrophobic hybrid nanowires composed of poly[bis(2,2,2-trifluoroethoxy)phosphazene]-Al_2_O_3_ (PTFEP/Al_2_O_3_) have been reported to show a topographic feature with a dual-scale roughness (micro and nano), forming a stable air cushion able to reduce the contact area between the surface and blood in contact below the liquid. A study is in progress to achieve 3D geometries for new coatings for cardiovascular devices (Scheme 17) [241,242].

**Figure 23:**
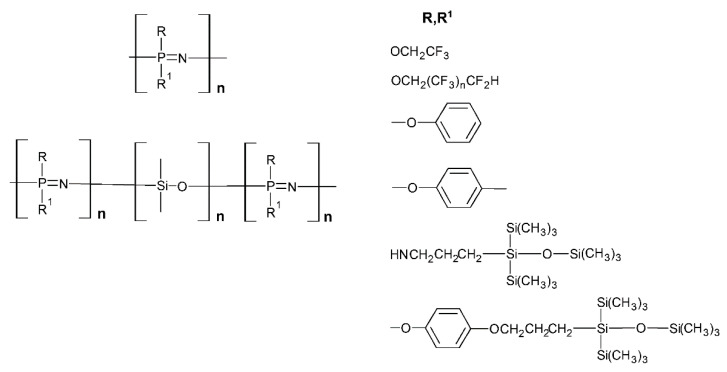
Hydrophobic polyphosphazenes.

Hydrophobic and superhydrophobic membranes by the casting or electrospinning of polyphosphazene fibers bearing the substituents reported in Figure 23 have been prepared: contact angles varied from 86° for the –[N=P(OCH_2_CF_3_)_2_]_x_–[Si(CH_3_)_2_–O]_y_ systems to 159° for the –[N=P(OCH_2_CF_3_)_2_]– one.
